# Unraveling the Bioactive Compounds and Multi‐Target Mechanisms of the Fructus Aurantii Immaturus‐Bambusae Caulis in Taeniam Herb Pair Against Chronic Gastritis: Integrating Identification of Absorbed Constituents, Targeted Network Pharmacology, and Computational Validation

**DOI:** 10.1002/fsn3.72016

**Published:** 2026-07-01

**Authors:** Yinghua Ma, Weiwei Xie, Jing Song, Yabin Qin, Yile Zhao

**Affiliations:** ^1^ Hebei Children's Hospital Shijiazhuang Hebei People's Republic of China; ^2^ Hebei Provincial Clinical Research Center for Child Health and Disease Shijiazhuang Hebei People's Republic of China; ^3^ The Second Hospital of Hebei Medical University Shijiazhuang Hebei People's Republic of China; ^4^ Hebei Provincial Hospital of Traditional Chinese Medicine Office of Drug Clinical Trial Institution Shijiazhuang Hebei People's Republic of China

**Keywords:** Bambusae Caulis in Taeniam, chronic gastritis, Fructus Aurantii Immaturus, molecular docking, molecular dynamics, targeted network pharmacology, UPLC‐Q‐TOF‐MS/MS

## Abstract

Chronic gastritis is a prevalent global health concern, and there is growing interest in functional foods and nutraceuticals derived from traditional herbs as complementary approaches for gastrointestinal health management. The Fructus Aurantii Immaturus‐Bambusae Caulis in Taeniam (FAI‐BCT) herb pair exhibits health‐promoting effects against chronic gastritis, but the underlying molecular mechanisms, particularly of its bioactive constituents, remain unclear. We established an ultra‐performance liquid chromatography coupled with quadrupole time‐of‐flight tandem mass spectrometry (UPLC‐Q‐TOF‐MS/MS) method to identify the blood‐absorbed constituents and metabolites of FAI‐BCT in rat plasma. Combined with GEO database mining and targeted network pharmacology, molecular docking and molecular dynamics simulations were further performed to explore its anti‐gastritis mechanisms. 27 prototype components and 13 metabolites were identified or preliminarily characterized in plasma. Targeted network pharmacology identified 3′,4′,5,7‐tetramethoxyflavone, 5′‐methoxynobiletin (M5), naringenin, hesperetin, p‐coumaric acid, ferulic acid, and nobiletin as key bioactive compounds. Core therapeutic targets included epidermal growth factor receptor (EGFR), signal transducer and activator of transcription 3 (STAT3), B‐cell lymphoma 2 (BCL2), matrix metalloproteinase‐9 (MMP9), protein kinase B‐α (AKT1), and prostaglandin‐endoperoxide synthase 2 (PTGS2). Gene Ontology (GO) and Kyoto Encyclopedia of Genes and Genomes (KEGG) enrichment analyses highlighted the PI3K‐Akt signaling pathway as a key mechanism regulated by these compounds. Molecular docking verified strong binding affinities, and molecular dynamics simulations validated stable complex formations. This study elucidates the multi‐target mechanisms of FAI‐BCT in preventing and managing chronic gastritis, emphasizing the role of its highly bioavailable bioactive constituents. The findings provide robust evidence supporting FAI‐BCT's potential application in functional foods or nutraceuticals for chronic gastritis and underscore the importance of bioavailability‐focused research on traditional Chinese medicine (TCM) constituents.

## Graphical abstract

This work systematically explored the anti‐chronic gastritis mechanism of the FAI‐BCT herb pair. UPLC‐Q‐TOF‐MS/MS was applied to identify absorbed prototypes and metabolites of FAI‐BCT in rat plasma. Combining database mining, network pharmacology, molecular docking, and molecular dynamics simulation, we screened the key bioactive components and core therapeutic targets of FAI‐BCT. This study reveals the multi‐component, multi‐target pharmacological characteristics of FAI‐BCT and provides a scientific basis for its development as functional foods or nutraceuticals against chronic gastritis.

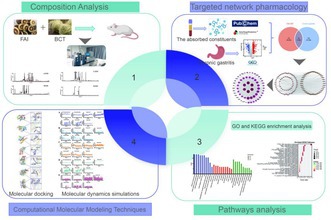

## 1 Introduction

Chronic gastritis is a global digestive system disorder whose pathogenesis is closely associated with *Helicobacter pylori* infection, autoimmune dysregulation, adverse dietary and lifestyle habits, and the use of nonsteroidal anti‐inflammatory drugs (NSAIDs). Long‐term progression may advance to chronic atrophic gastritis, subsequently inducing intestinal metaplasia and significantly elevating gastric cancer risk. In Western therapeutic strategies, eradication therapy is the mainstay for *Helicobacter pylori* ‐positive patients, yet it is increasingly challenged by emerging antibiotic resistance (Sugano et al. 2015; Correa and Piazuelo 2012). Symptomatic management predominantly relies on gastric mucosal protectants and acid‐suppressive agents, which often focus on symptom alleviation rather than addressing the root cause. By contrast, traditional Chinese medicine (TCM), grounded in the principle of “descending and harmonizing the stomach”, emphasizes the restoration of normal gastric physiological function. Its principles of “preventive treatment” and “preventing disease before its onset” align strongly with the imperative for preventing and managing gastric precancerous lesions. Numerous studies have confirmed the significant efficacy of TCM in reversing gastric precancerous lesions (Wang, Lian, et al. 2025; Yang et al. 2020).

Fructus Aurantii Immaturus (FAI) (Zhishi in Chinese), derived from the dried immature fruit of *Citrus aurantium L*. or its cultivated varieties (cv.), as well as *Citrus sinensis Osbeck (Rutaceae)*, possesses the pharmacological effects of dispersing qi stagnation, eliminating accumulation, resolving phlegm, and dissipating masses (Wu et al. 2022). In North America and Europe, FAI is commonly utilized for culinary purposes and as a functional nutritional supplement, while in Asian countries such as China and Japan, it is traditionally employed as an herbal remedy for gastric ulcers and dyspepsia (Liu et al. 2017; Tong et al. 2018). Bambusae Caulis in Taeniam (BCT) (Zhuru in Chinese), obtained from the dried middle layer of the stem of *Bambusa tuldoides Munr*o, *Sinocalamus beecheyanus* (Munro) *McClure var. pubescens P. F. Li*, or *Phyllostachys nigra (Lodd.) Munro var. henonis (Mitf.) Stapf ex Rendle (Poaceae)*, exhibits the efficacy of clearing heat, eliminating phlegm, harmonizing the stomach, and relieving restlessness and vomiting. Based on the traditional medicinal applications of BCT and its demonstrated anti‐inflammatory, antioxidant, and related pharmacological activities, China's National Health Commission has formally approved its use as both a Medicinal and Food Homologous Substance and functional food ingredient in accordance with national regulations (Lim et al. 2017). The combination of these two herbs boasts a long history of application, originating from the classical formula Wendan Tang (Gallbladder‐Warming Decoction) documented in Beiji Qianjin Yaofang (Essential Prescriptions Worth a Thousand Gold for Emergencies). The pair acts synergistically to strengthen the actions of harmonizing the stomach and descending counterflow, clearing heat and stopping vomiting, dispersing stagnation and transforming phlegm, and widening the chest to benefit the diaphragm. Preliminary studies indicate that FAI significantly reduces serum endotoxin and TNF‐α levels in rats, thereby mediating its antioxidant and anti‐inflammatory activities (Liu et al. 2017; He et al. 2025). Collaborative research has demonstrated that FAI effectively enhances gastrointestinal motility and alleviates functional dyspepsia through multi‐target mechanisms (Tan et al. 2017; Bai et al. 2018; Qiao et al. 2021). Its bioactive constituents, including flavonoids, coumarins, and volatile oils, have been validated as anti‐inflammatory agents (Lv et al. 2015). This suggests FAI holds potential as a preventative or therapeutic agent for chronic inflammatory disorders. Based on the traditional function of BCT in clearing stomach heat and arresting vomiting, the FAI‐BCT dyad holds promise for the clinical management of chronic gastritis and functional dyspepsia. Furthermore, the potential of the FAI‐BCT herb pair as either a dietary supplement or an herbal tea requires further exploration. Current research on FAI and BCT has predominantly focused on their chemical constituents in vitro. Conversely, studies investigating their combinatorial effects on in vivo constituents and underlying mechanisms remain largely unexplored.

Chemical constituents constitute the material basis for the therapeutic efficacy of TCM. Systematically elucidating the “absorption‐metabolism‐activity” trajectory of the chemical substance pool in an herbal pair has therefore become a core research proposition in the modernization of TCM. Currently, investigations into the bioactive substances of TCM have concentrated predominantly in vitro pharmacological mechanisms and the chemical constituents of constituents in crude materials. In contrast, the dynamic fate of these compounds after absorption and their metabolic transformation in vivo remain insufficiently elucidated. Given that the bioactive constituents of TCM often require in vivo biotransformation (e.g., intestinal flora metabolism, hepatic enzyme metabolism) to exert their pharmacological effects, direct analysis of the constituents within drug‐containing serum offers a more accurate reflection of the spectrum of pharmacodynamic substances directly acting within the body. This “Serum Pharmacochemistry” research strategy not only better aligns with the characteristic multi‐component, multi‐target, and holistic regulatory actions of TCM, but also embodies the holistic concept of TCM theory at the level of material basis research (Liu et al. 2023). Ultra‐high‐performance liquid chromatography coupled with quadrupole‐time‐of‐flight tandem mass spectrometry (UPLC‐Q‐TOF‐MS/MS), leveraging its ultra‐high resolution and high mass accuracy, has emerged as the preferred technique for the simultaneous characterization of prototype components and metabolites within complex TCM matrices (Xia et al. 2025; Wang, Chao, et al. 2025). This study employed UPLC‐Q‐TOF‐MS/MS to conduct comprehensive profiling and structural identification of the prototype constituents absorbed in vivo and their metabolites derived from the FAI‐BCT herb pair. This approach established a high‐confidence chemical space mapping the relationship between the “herb pair and its absorbed blood constituents”.

Recent advancements in biomedical big data and artificial intelligence have propelled the development of TCM network pharmacology. This methodology, by integrating computational simulations, experimental validation, and multi‐omics analyses, aligns well with the research of the macro‐regulatory mechanisms underlying the “drug‐target‐disease” relationships from the holistic perspective of TCM (Jiang et al. 2025; Wei et al. 2025). To circumvent the false‐positive targets frequently introduced by the over‐generalization of “virtual constituents” in conventional network pharmacology, we innovatively implemented a target‐centric network‐pharmacology strategy. A multi‐layer heterogeneous network encompassing “absorbed constituents‐direct targets‐pathways‐disease” was constructed, enabling cross‐scale mechanistic deconvolution from macroscopic wholism to microscopic molecules. Molecular docking predicts the binding mode and affinity between drug‐like small molecules and target proteins through computational simulation and remains a cornerstone in drug discovery and mechanism‐oriented research. Molecular dynamics simulation, performed at the atomic scale (simulation duration ≥ 100 ns), reveals the dynamic binding process of ligand‐receptor complexes, allowing the assessment of complex stability and key intermolecular interactions.

**FIGURE 1:**
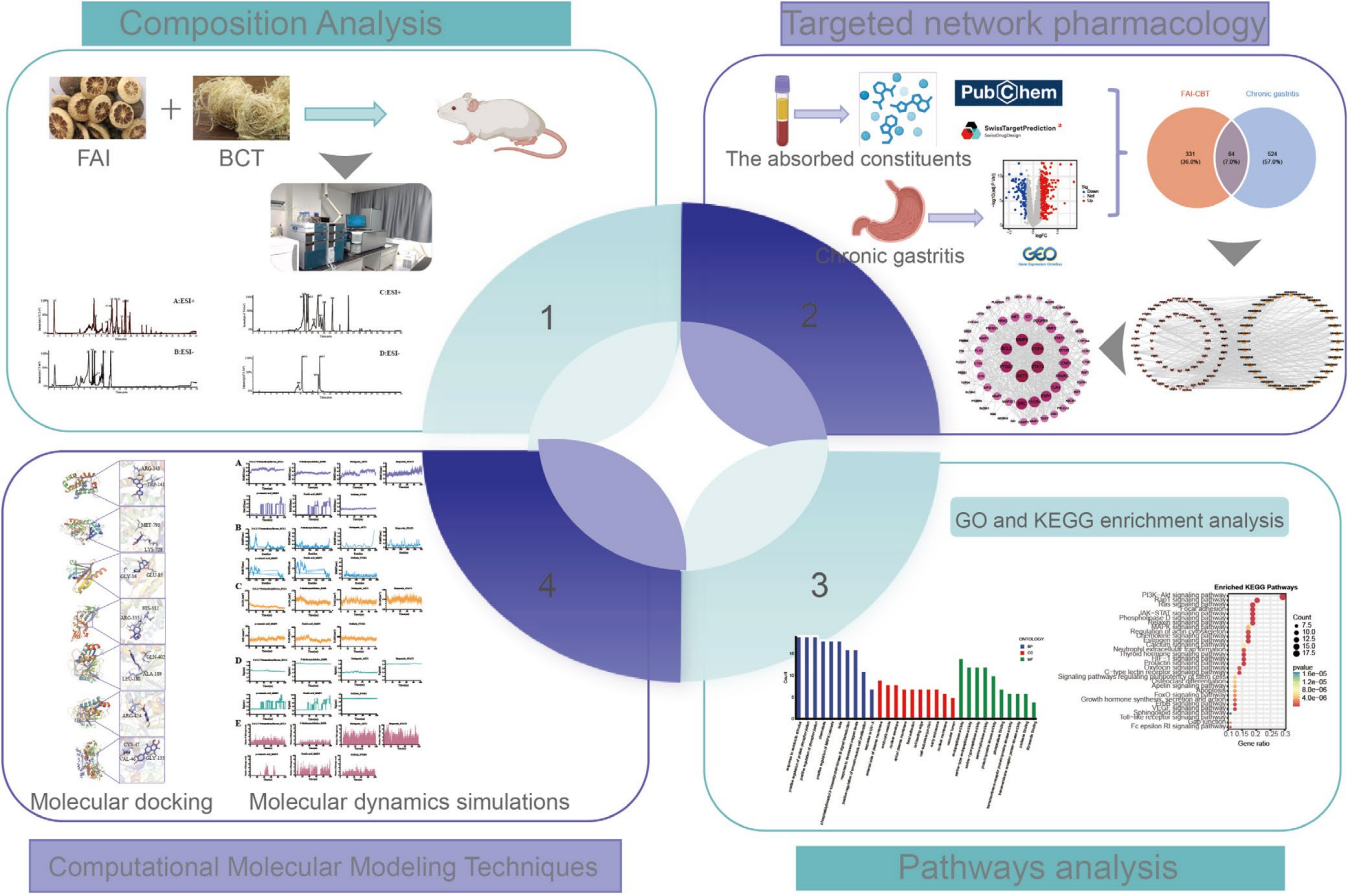
The complete research process.

This study integrates targeted network pharmacology, molecular docking, and molecular dynamics simulation methodologies to systematically decipher the molecular mechanisms by which the FAI‐BCT herb pair prevents and treats chronic gastritis. The complete research process is illustrated in Figure 1. This multi‐dimensional strategy aims to clarify both the pharmacodynamic substance basis and the biological underpinnings underlying the intervention against chronic gastritis, thereby providing theoretical foundations and informing design strategies for innovative TCM drug development.

## 2 Materials and Methods

### 2.1 Reagents and Materials

Acetonitrile and methanol were of LC/MS grade purchased from Thermo Fisher Scientific Co. Ltd. (China). The ultrapure water was purchased from Hangzhou Wahaha Group Co. Ltd. (Hangzhou, China). Formic acid was of LC/MS grade purchased from Mreda Technology Inc. (USA). The other reagents were of analytical grade. Hesperetin (JOT‐10356‐24,043,001), naringenin (JOT‐10030‐23,091,203), and narirutin (JOT‐10357‐24,042,401) were obtained from Chengdu Pufeide Biotechnology Co. Ltd. (Chengdu, PR China). The purity of the three substances was more than 99%. Nobiletin (Batch No.: 12055–202,102), neohesperidin (Batch No.: 111857–202,305), naringin (Batch No.: 110722–202,417), and ferulic acid (Batch No.: 110773–202,316) were purchased from National Institutes for Food and Drug Control (China). The purity of the four substances was more than 98%. Hesperidin (Batch No.: S61072156) and limonin (Batch No.: S312101826) were purchased from Shanghai Shifeng Biotechnology Co. Ltd. (Shanghai, China). The purity of hesperidin was more than 98%. P‐coumaric acid (Batch No.: B203354) was purchased from Shanghai Yuanye Biotechnology Co. Ltd. (Shanghai, China). The purity of p‐coumaric acid was more than 98%. Aurantium Fructus Immaturus (origin: Ganzhou, Jiangxi) and *Bambusa Tuldoides* Munro (origin: Bozhou, Anhui) were purchased from Bozhou Medicinal Materials Market (Anhui, China). The herbs were identified as the authentic Fructus Aurantii Immaturus and *Bambusa Tuldoides* Munro by Professor Wang Jianhua (Department of Pharmaceutical Analysis, School of Pharmacy, Hebei Medical University in Shijiazhuang, China). The specimens were deposited in the School of Pharmacy, Hebei Medical University. The medicinal materials were ground into powder and passed through a 60‐mesh sieve for further use.

### 2.2 Qualitative Analysis of FAI‐BCT

#### 2.2.1 Preparation of FAI‐BCT Extraction

FAI (10.0 g) and BCT (10.0 g) were mixed in a 1:1 ratio, then soaked in 200 mL of distilled water (1:10, *w/v*) for 0.5 h and refluxed for 1.5 h twice. The total extraction solutions were combined and concentrated under reduced pressure to obtain the intragastric administration solution of the herb pair (0.6 g·mL^−1^).

#### 2.2.2 Animals and Drug Administration

Six male Sprague–Dawley (SD) rats of SPF grade (weights 220 ± 20 g) were provided by the Laboratory Animal Center of Second Hospital of Hebei Medical University (Shijiazhuang, China, License number: SCXK (Ji) 2024–0001). The rats were housed in a suitable environment with a temperature of 20°C ± 2°C and 12 h light/dark cycles for 1 week and were allowed free access to food and water. All the animal experiments were approved by the Animal Ethics Committee of Research Ethics Committee of the Second Hospital of Hebei Medical University (2024‐AE065).

The rats were fasted for 12 h with free access to water denied prior to the experiment. Six rats were randomly divided into two groups: the control group (*n* = 3) and the FAI‐BCT group (*n* = 3). The herb extracts (6 g·kg^−1^) were administered to the FAI‐BCT group, while the control group was administered an equivalent volume of normal saline. The dosage of herbal medicine was calculated based on the daily clinical dosage in adults, and the rat dose of 6 g·kg^−1^ was finally converted by body surface area normalization.

#### 2.2.3 Plasma Collection and Preparation

All rats were subjected to blood collection from the retro‐orbital venous plexus at 0.25, 0.5, 1, 2, 4, 8, 12, and 24 h after drug administration. The blood samples (0.5 mL each) were collected into EP tubes containing 1% sodium heparin. The samples were then centrifuged at 3000 rpm for 5 min at 4°C, and the plasma was obtained. Plasma samples from the same group at different time points were pooled into a single sample and stored at −80°C for further analysis. The collection of blank plasma was performed in the same manner as for the experimental group. For sample preparation, 50 μL of plasma was mixed with 150 μL of methanol, vortexed for 2 min, and then centrifuged at 12000 rpm for 15 min at 4°C. The supernatant was transferred to a new EP tube. This extraction procedure was repeated three times, and the combined supernatants were evaporated under a nitrogen stream at 37°C. The residue was reconstituted in 100 μL of 95% acetonitrile, vortexed for 5 min, and centrifuged at 12000 rpm for 10 min. The supernatant was then subjected to UPLC‐Q‐TOF‐MS/MS analysis.

#### 2.2.4 UPLC‐Q‐TOF‐MS/MS Conditions

The UPLC‐Q‐TOF‐MS/MS consisted of a 40DX3 UPLC System (Shimadzu, Japan) and a X500R Quadrupole TOF system (AB SCIEX, USA) equipped with Duo‐Spray ion sources in the electrospray ionization (ESI) technology. The chromatographic column used was a Phenomenex Luna Omega Polar C_18_ column (2.1 mm × 100 mm, 1.6 μm). The mobile phase consisted of 0.1% formic acid in water (A) and acetonitrile (B), with a flow rate of 0.3 mL·min^−1^. The gradient elution conditions were set as follows: 0 ~ 2 min, 5% B; 2 ~ 4 min, 5% ~ 20% B; 4 ~ 18 min, 20% ~ 95% B; 18 ~ 20 min, 95% B; 20 ~ 20.1 min, 95% ~ 5% B; 20.1 ~ 25 min, 5% B. The injection volume was 10 μL, and the column temperature was maintained at 35°C. The mass spectrometer utilized was a time‐of‐flight mass spectrometer (TOF‐MS) with electrospray ionization in positive or negative ion switching mode. The data acquisition mode was high‐resolution full scan/ion‐dependent acquisition (TOF MS ~ Product Ion~IDA). The 15 most intense peaks with a response value exceeding 100 cps were simultaneously subjected to second‐order mass spectrometry scanning, with dynamic background subtraction (DBS) enabled. The optimized mass spectrometry parameters were as follows: ion source turbo spray temperature, 550°C; nebulizer gas (Gas 1), 50 psi; heater gas (Gas 2), 50 psi; curtain gas (CUR), 25 psi; total scan time, 0.643 s; and data acquisition range, 50 ~ 1000 Da. In positive ion mode, the source spray voltage was 5500 V, declustering potential (DP) was 50 V, collision energy (CE) was 35 V, and collision energy spread (CES) was 15 V. In negative ion mode, the source spray voltage was −4500 V, DP was −80 V, CE was −35 V, and CES was 15 V.

#### 2.2.5 Data Analysis Strategy

Based on the Analytics functionality within the SCIEX OS system, comparisons and analyses were conducted among drug‐administered plasma, blank plasma, and herbal extract solutions. The original data were processed in the SCIEX OS system's Analytics software for component screening and preliminary identification, using reference standards information and the natural compound small molecule library (provided by SCIEX). The identification criteria were based on the “traffic light” filtering function, with the following settings: a deviation of less than ±10 ppm for the first‐order mass‐to‐charge ratio, a deviation of less than ±5 ppm for the isotopic abundance ratio, and a spectral library match score greater than 70%. If all three conditions were met, it was marked as a green light, indicating high‐confidence positive confirmation. For compounds with reference standards, confirmation was based on chromatographic retention time and first‐order and second‐order mass spectrometry data. For compounds without reference standards, identification was inferred based on the self‐built database, isotopic information, characteristic ion fragments, and the reported cleavage patterns of related components in the literature. The strategy for identifying metabolites is as follows: (1) The Molecule Profiler software was used to rapidly and accurately identify metabolites, employing functions to process collision‐induced dissociation (CID) and electron‐activated dissociation (EAD) data for reliable biotransformation and metabolite structural identification. (2) The Analytics software was used to compare chromatographic peaks between the blank and experimental groups, screening for peaks present in the experimental group but absent in the blank group. (3) The important parameter Clog P in ChemDraw 19.0 and retention time trends was utilized to identify isomers of metabolites. Typically, compounds with higher Clog P values exhibit longer retention times on reversed‐phase chromatographic columns.

#### 2.2.6 Qualitative Method Validation

Comprehensive validation of the qualitative analysis for the absorbed prototype components of FAI‐BCT drug pair in plasma was performed in accordance with the guidelines issued by the U.S. Food and Drug Administration/International Council for Harmonization (FDA/ICH), with emphasis on specificity, precision, matrix effect, and stability. Six batches of blank rat plasma were employed as the matrix, and quality control (QC) samples at known concentrations were prepared using reference standards including p‐coumaric acid, ferulic acid, naringenin, hesperetin, and nobiletin at known concentrations. (1) Specificity: The absence of interference from the plasma matrix on mass spectrometric identification of the absorbed components was verified by comparing the chromatograms of blank plasma, reference standard solution, blank plasma spiked with standards (QC samples), and drug‐containing plasma. (2) Precision: Within‐run precision was evaluated by analyzing six replicate QC samples, while between‐run precision was assessed via three consecutive validation batches over 3 days. The acceptance criteria for precision were defined as follows: relative standard deviation (RSD) of retention time ≤ 0.5% (indicating chromatographic system stability), RSD of peak area ≤ 15% (reflective of mass spectrometric response stability), and mass accuracy deviation ≤ 5 ppm. (3) Matrix effect: It was investigated by comparing the peak areas of reference compounds in blank plasma spiked samples against those in pure methanol solutions. (4) Stability: Stability profiles of QC samples were evaluated under the following conditions: storage at room temperature for 4 h, refrigeration at 4°C for 12 h, three freeze–thaw cycles, post‐preparation storage in the autosampler at 4°C for 24 h, and storage at −80°C for 21 days.

### 2.3 Target Network Pharmacology

#### 2.3.1 Identification of Active Ingredients in FAI‐BCT and Target Prediction

The prototype constituents and metabolites identified by UPLC‐Q‐TOF‐MS/MS were used to build the chemical compound library of FAI‐BCT for subsequent targeted network pharmacology research. The SMILES information of these chemical constituents was retrieved from the PubChem database. Subsequently, the targets of these compounds were predicted using their SMILES information via the Swiss Target Prediction (http://www.swisstargetprediction.ch/), applying a probability threshold of ≥ 0.05.

#### 2.3.2 GEO Differential Gene Analysis and Screening of Chronic Gastritis Targets

Gene expression datasets associated with chronic gastritis were retrieved from the Gene Expression Omnibus (GEO; https://www.ncbi.nlm.nih.gov/geo/). The screening criteria for GEO datasets were defined as follows: datasets were required to enroll both chronic gastritis patients and healthy controls, and only mRNA expression profiles (microarray/RNA‐seq) were included while non‐mRNA data were excluded. Each group contained no fewer than 3 samples to ensure statistical reliability. Datasets with complete original expression data, detailed clinical information and standard case–control design were retained, whereas those with incomplete data, low quality or mixed disease types were eliminated. Ultimately, four qualified datasets including GSE233973, GSE60427, GSE27411 and GSE2669 were selected for further analyses. Raw gene expression data were integrated and subjected to batch effect correction using the “limma” and “sva” packages in R software (version 4.4.2). Principal Component Analysis (PCA) was performed to visualize sample distribution patterns post‐correction. Differentially expressed genes (DEGs) were identified with limma under the criteria |logFC| > 1 and *p*‐value < 0.05. Volcano plots and heatmaps were generated using “ggplot2” and “pheatmap” to display the DEG profiles. In parallel, chronic gastritis‐related targets were systematically curated from established gene and drug databases, including GeneCards (https://www.genecards.org/), OMIM (https://omim.org/), TTD (http://db.idrblab.net/ttd/), PharmGKB (https://www.pharmgkb.org/), and DrugBank (https://go.drugbank.com/). The curated gene lists from these databases were then merged with the DEGs obtained from GEO, followed by deduplication, to yield a final set of disease‐relevant targets.

#### 2.3.3 Compound‐Target Interaction Network and Screening of Core Ingredients

The intersection between targets of active ingredients in FAI‐BCT and targets associated with chronic gastritis was obtained using bioconductor packages in R software (version 4.4.2). The compound‐target interaction network was then constructed using Cytoscape version 3.7.2. Compounds with a degree value ≥ 10 were selected as the potential active constituents of the FAI‐BCT for intervening in chronic gastritis.

#### 2.3.4 PPI Network, GO and KEGG Enrichment Analysis

Protein–protein interaction (PPI) network was established using the STRING database, restricting the analysis to *Homo sapiens* (human) interactions with the highest confidence threshold (interaction score > 0.90). Perl scripts were employed to generate the network and type files, which were subsequently imported into Cytoscape for visualization and topological analysis. Targets with a degree value ≥ 10 were defined as core targets. Gene Ontology (GO) enrichment analysis and Kyoto Encyclopedia of Genes and Genomes (KEGG) pathway analysis were performed on the intersecting targets using R software with bioconductor environment. This resulted in a bar plot for the GO enrichment analysis, and bubble plots and pathway maps for the KEGG pathway analysis, depicting terms/pathways with a significance threshold of *p*‐value < 0.05.

### 2.4 Molecular Docking and Molecular Dynamics

#### 2.4.1 Molecular Docking

Molecular docking, a computer‐aided drug‐design technique, was employed to identify the optimal conformation and the most stable binding affinity between ligands and receptors. A lower calculated binding free energy indicates a more stable binding affinity [Hu et al. 2024; Zhang et al. 2024]. To validate interactions and determine binding affinity, molecular docking was performed between the screened core compounds and core target proteins. Chemical structures of the core compounds were retrieved from the PubChem (https://pubchem.ncbi.nlm.nih.gov/) database, while 3D structures of the core targets were obtained from the RCSB PDB (https://www.rcsb.org/) database. After importing these compounds and protein structures into Auto Dock Tools (version 1.5.6), the files were optimized and saved in “pdbqt” format. The active site of each protein was defined, and docking calculations were executed using Auto Dock Vina. Visualization of the docking results was performed using Pymol software (version 2.5).

#### 2.4.2 Molecular Dynamics Simulations

Based on the protein‐ligand complex structures obtained by molecular docking, molecular dynamics simulations were performed using GROMACS 2023.2 to thoroughly evaluate the binding stability and dynamic interactions of each complex [Li et al. 2025]. The topology files for the protein and the ligand were prepared separately: the protein was described using the Amber 99SB force field combined with the TIP3P water model, while the ligand topology was generated with Sobtop 1.0 under consistent force field parameters. The initial coordinate files (.gro) and topology files (.top and .itp), which include force field and topological information, were obtained for each component. The topology and structure files of the protein and ligand were merged, and the resulting complex was subjected to periodic solvation. This process involved defining the simulation box, adding TIP3P water molecules, and introducing ions to neutralize the system charge. Energy minimization was then carried out to relieve steric clashes and unreasonable conformations. Position restraints were applied to the protein‐ligand complex to maintain the initial binding pose during initial equilibration. Finally, a 100 ns molecular dynamics simulation was conducted under the NPT ensemble. Throughout the simulation, time‐dependent changes of key physicochemical properties were monitored, including the root mean square deviation (RMSD), root mean square fluctuation (RMSF), radius of gyration (Rg), solvent accessible surface area (SASA), and the number of hydrogen bonds (NHB). These metrics were visualized to systematically assess the structural stability and binding behavior of the complex in a dynamic environment.

## 3 Results and Discussion

### 3.1 Qualitative Analysis

**FIGURE 2:**
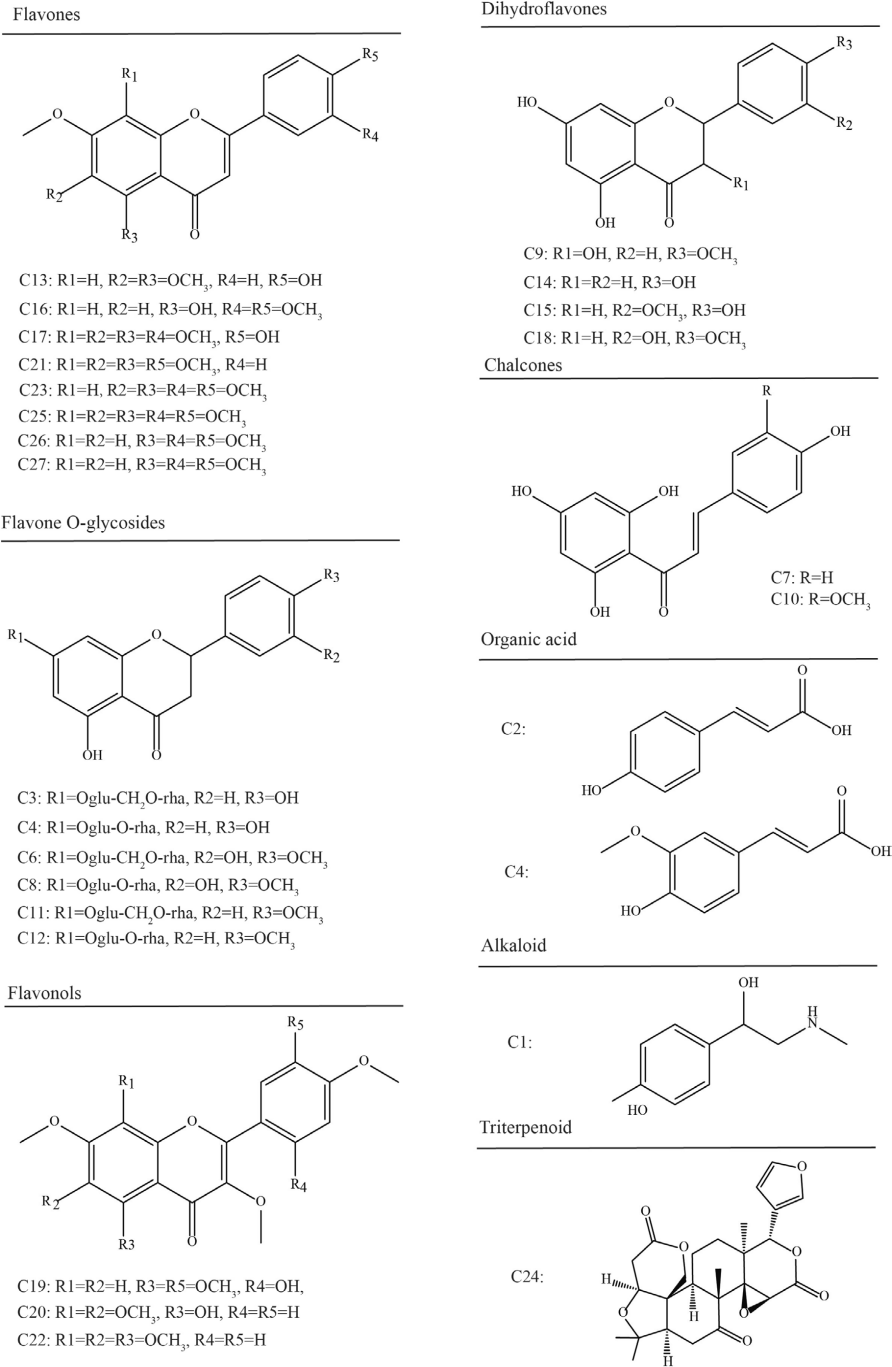
Chemical structures of the absorbed prototypes and metabolites of FAI‐BCT herb pair.

**FIGURE 3:**
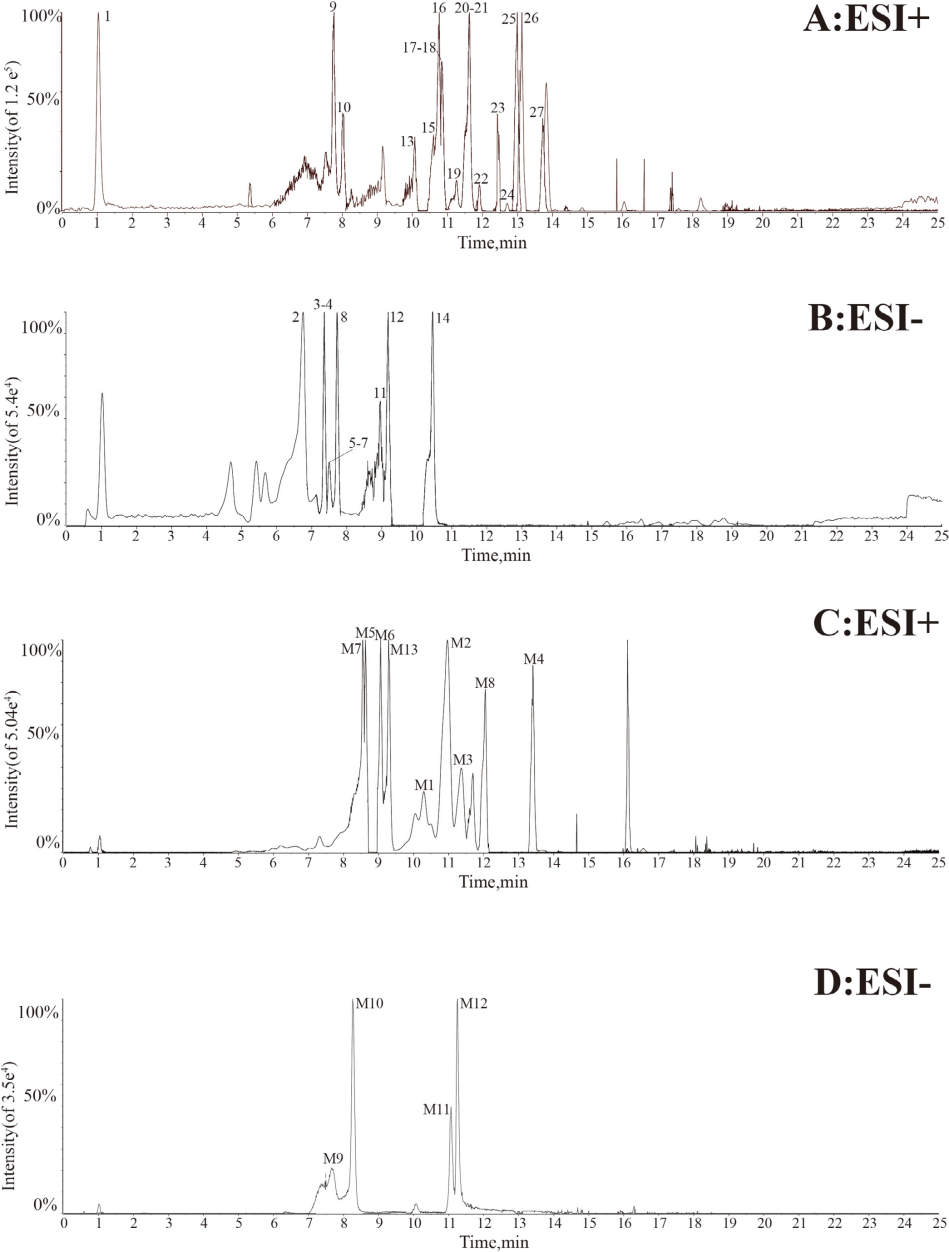
Total ion chromatogram (TIC) of prototype components and metabolites in rat plasma after oral administration of FAI‐BCT herb pair: (A) prototype components in positive ion mode; (B) prototype components in negative ion mode; (C) metabolites in positive ion mode; (D) metabolites in negative ion mode.

A total of 27 prototype constituents were identified in rat plasma following oral administration of the FAI‐BCT combination. These were assigned by matching retention characteristics, mass‐spectral fragmentation patterns, accurate masses and MS/MS fragment ions with those of reference standards or literature data. These included 23 flavonoids, 1 alkaloid, 2 organic acids, and 1 triterpenoid. In addition, 13 metabolites entering the bloodstream were characterized. The chromatographic and fragment ion data of prototype constituents are summarized in Table 1, the structural formulas of prototypes are depicted in Figure 2, and the extracted ion chromatogram (XIC) of both prototypes and metabolites in both positive‐ and negative‐ion modes are presented in Figure 3.

**TABLE 1:** Characterization of the absorbed prototype constituents in dosed plasma by UPLC‐Q‐TOF‐MS/MS.

No.	t_R_/min	Formula	Adduct	Calculated mass	Measured mass	Error/ppm	MS/MS fragments	Identification	Source
**C1**	1.03	C_9_H_13_NO_2_	[M + H]^+^	168.1019	168.1020	0.59	150.0908, 135.0673, 119.0495, 107.0489, 91.0537, 77.0385	Synephrine	FAI
**C2**	6.65	C_9_H_8_O_3_	[M − H]^−^	163.0401	163.0404	1.84	119.0502, 117.0343, 93.0343, 65.0403	p‐coumaric acid^a^	BCT
**C3**	7.13	C_27_H_32_O_14_	[M − H]^−^	579.1719	579.1725	1.04	271.0620, 151.0056	Narirutin^a^	FAI
**C4**	7.03	C_10_H_10_O_4_	[M − H]^−^	193.0506	193.0513	3.63	178.0272, 1330.0292, 89.0396	Ferulic acid^a^	BCT
**C5**	7.36	C_27_H_32_O_14_	[M − H]^−^	579.1719	579.1725	1.04	459.1165, 339.0760, 271.0620, 151.0046	Naringin^a^	FAI
**C6**	7.51	C_28_H_34_O_15_	[M − H]^−^	609.1825	609.1832	1.15	343.0916, 301.0728	Hesperidin^a^	FAI
**C7**	7.57	C_15_H_12_O_5_	[M − H]^−^	271.0612	271.0619	2.58	139.0742	Naringenin chalcone	FAI
**C8**	7.73	C_28_H_34_O_15_	[M − H]^−^	609.1825	609.1833	1.31	343.0850, 301.0724, 286.0497	Neohesperidin^a^	FAI
**C9**	7.75	C_16_H_14_O_6_	[M + H]^+^	303.0863	303.0868	1.65	177.0554, 153.0197, 137.0616	3,5,7‐trihydroxy‐4′‐methoxyflavanone	FAI
**C10**	8.02	C_16_H_14_O_6_	[M + H]^+^	303.0863	303.0869	1.98	177.0535, 153.0191	Homoeriodictyol chalcone	FAI
**C11**	8.95	C_28_H_34_O_14_	[M − H]^−^	593.1876	593.1898	3.71	512.2716, 285.0775	Didymin	FAI
**C12**	9.18	C_28_H_34_O_14_	[M − H]^−^	593.1876	593.1887	1.85	285.0797	Poncirin	FAI
**C13**	10.06	C_18_H_16_O_6_	[M + H]^+^	329.1020	329.1027	2.13	314.0763, 299.0595, 271.0619, 181.0153, 153.0199	4′‐Hydroxy‐5,6,7‐trimethoxyflavone	FAI
**C14**	10.27	C_15_H_12_O_5_	[M − H]^−^	271.0612	271.0615	1.11	187.0411, 177.0213, 151.0042, 119.0504, 65.0034	Naringenin^a^	FAI
**C15**	10.65	C_16_H_14_O_6_	[M + H]^+^	303.0863	303.0873	3.30	153.0197	Homoeriodictyol	FAI
**C16**	10.75	C_18_H_16_O_6_	[M + H]^+^	329.1020	329.1026	1.82	314.0798, 299.0561, 285.0774, 268.0741, 153.0198	5‐Hydroxy‐3′,4′,7‐trimethoxyflavone	FAI
**C17**	10.82	C_20_H_20_O_8_	[M + H]^+^	389.1231	389.1235	1.03	374.1020, 359.0798	4′‐hydroxy‐3′,5,6,7,8‐pentamethoxyflavone	FAI
**C18**	10.64	C_16_H_14_O_6_	[M + H]^+^	303.0863	303.0867	1.32	177.0549, 153.0194, 145.0315	Hesperetin^a^	FAI
**C19**	11.26	C_20_H_20_O_8_	[M + H]^+^	389.1231	389.1235	1.03	359.0781, 159.1176	2′‐Hydroxy‐3,5,7,4′,5′‐pentamethoxyflavone	FAI
**C20**	11.62	C_20_H_20_O_8_	[M + H]^+^	389.1231	389.1227	−1.03	374.1029, 59.0779, 353.0506, 335.2384, 157.1038	5‐Hydroxy‐3,6,7,8,4′‐pentamethoxyflavone	FAI
**C21**	11.63	C_20_H_20_O_7_	[M + H]^+^	373.1282	373.1282	0.00	355.2640, 343.0843, 319.2445, 241.1954, 173.1325, 159.1180, 133.1008, 121.1010	Tangeretin	FAI
**C22**	11.97	C_21_H_22_O_8_	[M + H]^+^	403.1387	403.1396	2.23	388.1167, 373.0912, 358.0717, 345.0995, 327.0874, 317.1024, 165.0564	3‐Methoxytangeretin	FAI
**C23**	12.30	C_20_H_20_O_7_	[M + H]^+^	373.1282	373.1251	−8.31	357.0944, 343.0785, 328.0579, 179.0345, 153.0180	Sinensetin	FAI
**C24**	12.40	C_26_H_30_O_8_	[M + H]^+^	471.2013	471.2009	−0.85	453.1915, 435.1816, 425.1937, 407.1864, 367.1901, 339.0964, 321.1857, 279.1395, 253.1242, 213.0919, 187.0767, 161.0592, 133.0659, 119.0877, 105.0708, 95.0135, 81.0352, 69.0714	Limonin^a^	FAI
**C25**	12.85	C_21_H_22_O_8_	[M + H]^+^	403.1387	403.1391	0.99	388.1178, 373.0924, 355.0852, 327.0884, 301.0683	Nobiletin^a^	FAI
**C26**	13.12	C_19_H_18_O_6_	[M + H]^+^	343.1176	343.1179	0.87	328.0844, 313.0679, 299.0893, 153.0182	3′,4′,5,7‐Tetramethoxyflavone	FAI
**C27**	13.72	C_20_H_20_O_7_	[M + H]^+^	373.1282	373.1285	0.80	355.2646, 343.0829, 315.0884, 261.1894	Isosinensetin	FAI

Abbreviations: BCT, Bambusae Caulis in Taeniam; FAI, Fructus Aurantii Immaturus.

^a^ Compounds validated by reference substances.

#### 3.1.1 Identification of Prototypes of FAI‐BCT in Rat Plasma

**FIGURE 4:**
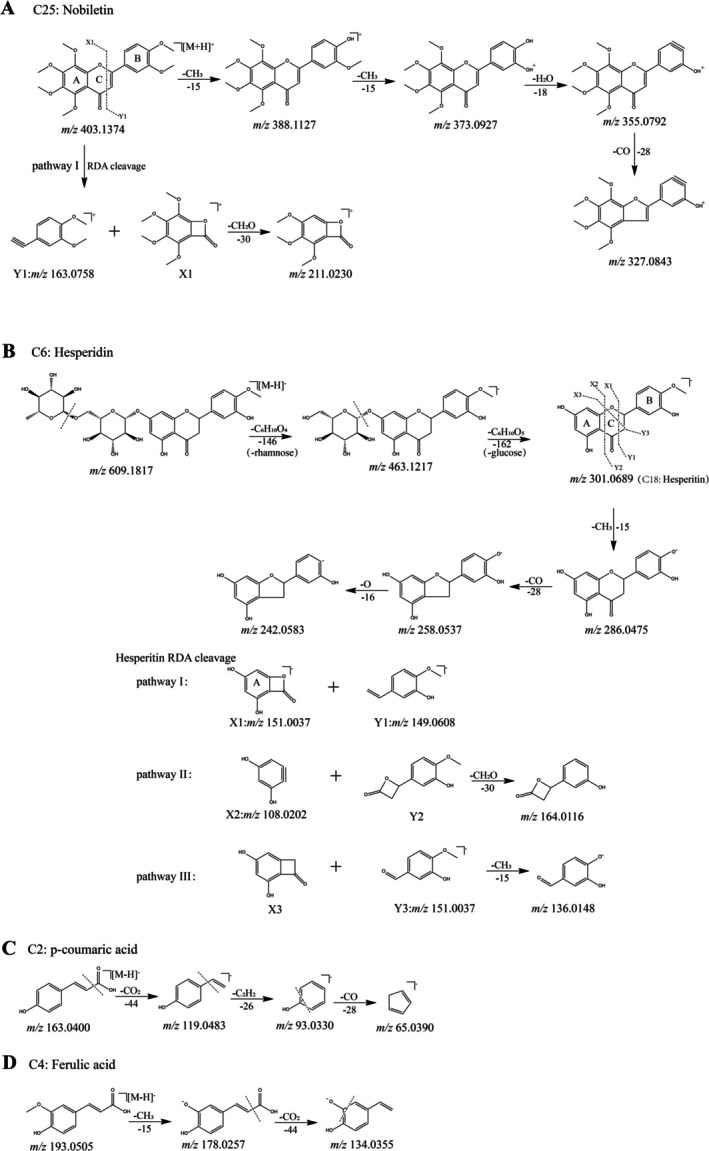
MS_2_ spectrum and fragmentation pathways of representative prototype components: (A) C25‐nobiletin; (B) C6‐hesperidin and C18‐hesperetin; (C) C2‐p‐coumaric acid; (D) C4‐ferulic acid.

Among the constituents absorbed into the bloodstream from FAI‐BCT, flavonoids were identified as the major components. These typically exist as glycosides or aglycones, all sharing a similar core scaffold. The flavonoids detected in the bloodstream in this study were broadly categorized into five classes: flavones, dihydroflavones, flavonols, chalcones, and flavone O‐glycosides. Flavonoids identified in this study‐namely nobiletin, hesperetin, naringenin, narirutin, hesperidin, naringin, and neohesperidin‐were unambiguously confirmed by comparing their chromatographic retention times and primary/secondary mass spectrometry (MS/MS) data with authentic reference standards. MS fragmentation of flavonoid O‐glycosides is initiated by the sequential loss of monosaccharide residues (e.g., rhamnose, −146 Da; glucose, −162 Da) to afford the corresponding aglycone ion. Subsequent fragmentation of the aglycone proceeds via consecutive eliminations of small neutral molecules such as H_2_O, CH_3_, or CO, and by Retro‐Diels‐Alder (RDA) rearrangements within the C‐ring. Three principal RDA cleavage patterns were observed (details in Figure 4). Compound 25, representative of flavones, was exhibited an accurate [M + H]^+^ ion at *m/z* 403.1374 with a mass error of −0.50 ppm (< ±10 ppm) in positive ion mode. Diagnostic product ions were detected at *m/z* 388.1127 ([M + H‐CH_3_]^+^), 373.0927 ([M + H‐2CH_3_]^+^), 355.0792 ([M + H‐2CH_3_‐H_2_O]^+^), and 327.0843 ([M + H‐2CH_3_‐H_2_O‐CO]^+^). RDA fragmentation of the C‐ring produced fragments X1 and Y1 (*m/z* 163.0758), with ion X1 subsequently losing CH_2_O to yield *m/z* 211.0230. The proposed fragmentation pathway is detailed in Figure 4A. Based on the retention time of 12.85 min, consistent with that of the reference standard, compound 25 was identified as nobiletin. Compound 6 (hesperidin) and compound 18 (hesperetin) were analyzed as representatives of flavonoid glycosides and dihydroflavones, respectively. In negative ion mode, compound 6 exhibited a quasi‐molecular ion peak [M‐H]^−^ at *m/z* 609.1817 (mass error: 1.15 ppm, < ±10 ppm). Loss of rhamnose generated the ion at *m/z* 463.1217, and subsequent loss of glucose afforded the aglycone ion at *m/z* 301.0689 (compound 18: hesperetin). This aglycone then fragmented according to the characteristic pattern of hesperetin: sequential losses of CH_3_, CO, and O produced ions at *m/z* 286.0475, 258.0537, and 242.0583, respectively. Three RDA cleavage modes were observed: Pathway I: fragments X1 (*m/z* 151.0037) and Y1 (*m/z* 149.0608); Pathway II: fragments X2 (*m/z* 108.0202) and Y2, Y2 subsequently lost CH_2_O to give *m/z* 164.0116; Pathway III: fragments X3 and Y3 (*m/z* 151.0037), Y3 then lost CH_3_ to yield *m/z* 136.0148. The proposed fragmentation pathways are illustrated in Figure 4B. Compounds 6 and 18 eluted at 7.51 min and 10.64 min (negative mode), respectively, matching the retention times of authentic hesperidin and hesperetin standards, and were thus confirmed as hesperidin and hesperetin.

Two organic acid constituents in the FAI‐BCT formula absorbed into the bloodstream were unambiguously identified by comparison with reference standards: p‐coumaric acid (Compound 2) and ferulic acid (Compound 4). Compound 2 exhibited a deprotonated molecule ion [M‐H]^−^ at *m/z* 163.0400 in negative ion mode, with a mass error of 1.84 ppm (within ±10 ppm). Subsequent fragmentation involved the consecutive losses of CO_2_ (−44 Da), C_2_H_2_ (−26 Da) and CO (−28 Da) to generate product ions at *m/z* 119.0483, 93.0330 and 65.0390, respectively. Based on the comparison of the secondary ion fragments and retention time with the p‐coumaric acid reference standard, Compound 2 was confirmed to be p‐coumaric acid. The proposed fragmentation pathway is illustrated in Figure 4C. Compound 4 displayed a deprotonated molecular ion [M‐H]^−^ at *m/z* 193.0505 in negative ion mode, with a mass deviation of 3.63 ppm (within ±10 ppm). The ion at *m/z* 178.0257 corresponded to the loss of a methyl radical CH_3_, (−15 Da), followed by the expulsion of CO_2_ (−44 Da) to yield *m/z* 134.0355. The retention time of Compound 4 in negative‐ion mode was 7.03 min, identical to that of the ferulic acid reference standard, thereby identifying Compound 4 as ferulic acid. The proposed fragmentation pathway is illustrated in Figure 4D.

Other constituents, such as the alkaloid synephrine and the triterpenoid limonin, were identified by matching their retention times and secondary ion fragments with those of the corresponding reference standards.

#### 3.1.2 Identification of Metabolites of Typical Constituents in Rat Plasma

**FIGURE 5:**
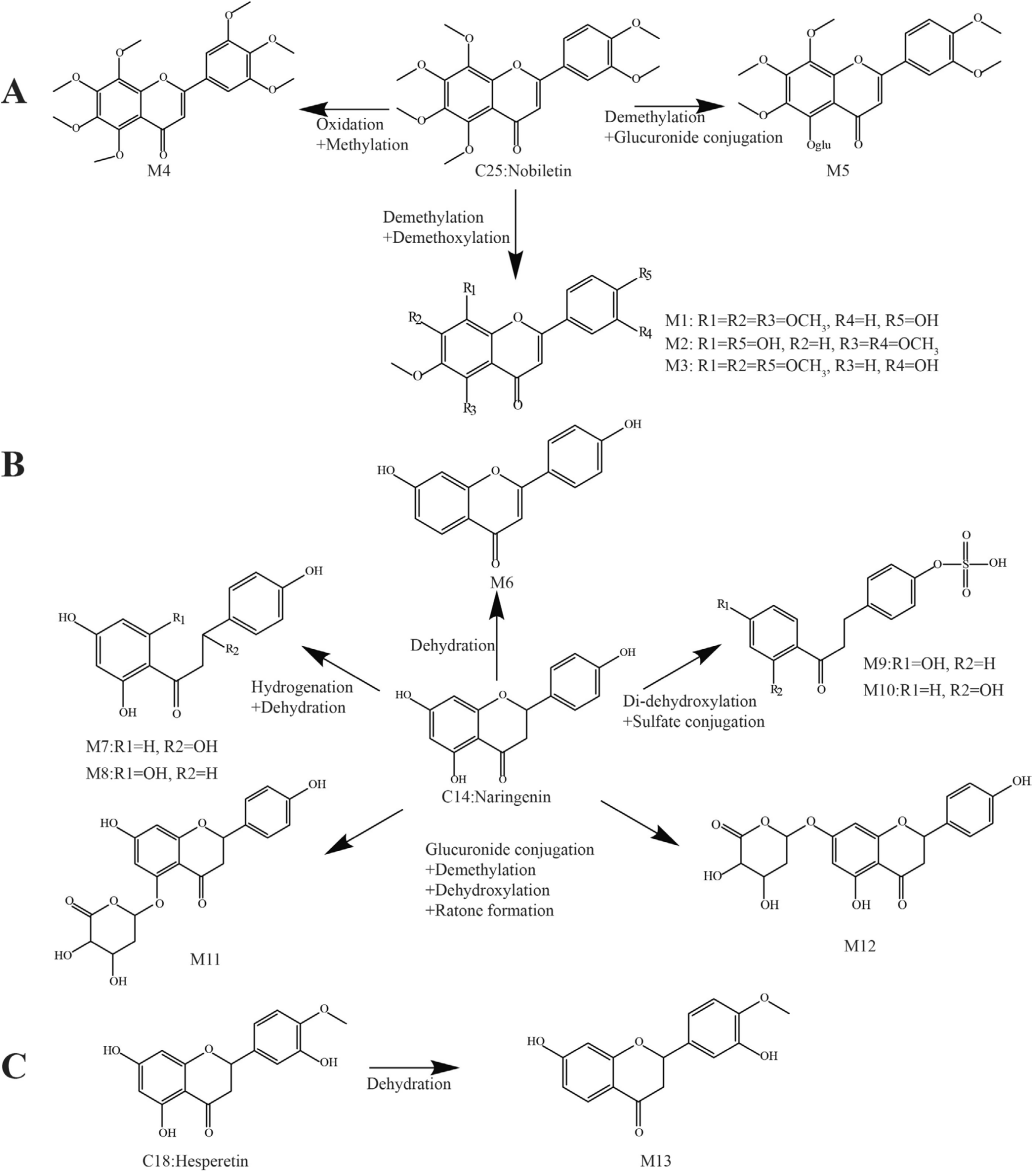
Metabolic profile and proposed metabolic pathways of representative prototype components: (A) C25‐nobiletin; (B) C14‐naringenin; (C) C18‐hesperetin.

In this study, flavonoids were identified as the predominant absorbed constituents and the principal bioactive components of the FAI‐BCT combination. Therefore, nobiletin, naringenin, and hesperetin were selected as representative markers to profile the metabolites generated from the absorbed constituents. Phase I metabolic transformations include dehydroxylation, demethylation, oxidation, demethoxylation, and hydrogenation; Phase II metabolic transformations encompass methylation, glucuronidation, and sulfation. A total of 13 metabolites were characterized in the plasma. The chromatographic and fragment ion data of metabolites are summarized in Table 2. The chemical structures of these metabolites and proposed metabolic pathways are delineated in Figure 5.

**TABLE 2:** Characterization of the metabolites in dosed plasma by UPLC‐Q‐TOF‐MS/MS.

No.	t_R_/min	Formula	Adduct	Calculated mass	Measured mass	Error/ppm	MS/MS fragments	Composition shift
**M1**	10.29	C_19_H_18_O_7_	[M + H]^+^	359.1125	359.1130	1.39	329.0674, 301.0728	C25 + Demethylation + Demethoxylation
**M2**	10.97	C_19_H_18_O_7_	[M + H]^+^	359.1125	359.1128	0.84	329.0639, 315.0870, 301.0723, 298.0852	C25 + Demethylation + Demethoxylation
**M3**	11.37	C_19_H_18_O_7_	[M + H]^+^	359.1125	359.1132	1.95	329.0655	C25 + Demethylation + Demethoxylation
**M4**	13.40	C_22_H_24_O_9_	[M + H]^+^	433.1493	433.1497	0.92	403.1018, 373.0808	C25 + Oxidation + Methylation
**M5**	8.63	C_26_H_28_O_14_	[M + H]^+^	565.1552	565.1558	1.04	389.1244, 359.0784	C25 + Demethylation + Glucuronide conjugation
**M6**	9.06	C_15_H_10_O_4_	[M + H]^+^	255.0652	255.0655	1.18	237.0569, 227.0714, 199.0771, 181.0668, 137.0245	C14 + Dehydration
**M7**	8.55	C_15_H_16_O_4_	[M + H]^+^	261.1121	261.1126	1.91	243.1072	C14 + Hydrogenation + Dehydration
**M8**	12.03	C_15_H_16_O_4_	[M + H]^+^	261.1121	261.1126	1.91	243.1042	C14 + Hydrogenation + Dehydration
**M9**	7.66	C_15_H_14_O_6_S	[M − H]^−^	321.0438	321.0446	2.49	241.0901, 135.0459, 121.0298	C14 + Di‐dehydroxylation + Sulfate conjugation
**M10**	8.27	C_15_H_14_O_6_S	[M − H]^−^	321.0438	321.0441	0.93	241.0878, 135.0451, 121.0296, 79.9576	C14 + Di‐dehydroxylation + Sulfate conjugation
**M11**	11.07	C_20_H_18_O_9_	[M − H]^−^	401.0878	401.0885	1.75	357.0638, 313.0709, 269.0825	C14 + Glucuronide conjugation + Demethylation + Dehydroxylation + Ratone formation
**M12**	11.25	C_20_H_18_O_9_	[M − H]^−^	401.0878	401.0880	0.50	357.0594, 313.0727, 269.0828	C14 + Glucuronide conjugation + Demethylation + Dehydroxylation + Ratone formation
**M13**	9.29	C_16_H_12_O_5_	[M + H]^+^	285.0757	285.0762	1.75	270.0539, 242.0591, 225.0562	C18 + Dehydration

##### 3.1.2.1 Metabolites of Nobiletin

The major biotransformation reactions of nobiletin metabolites include demethylation, demethoxylation, oxidation, methylation, and glucuronidation. Metabolites M1, M2, and M3, with the molecular formula C_19_H_18_O_7_ , are 44 Da lower than nobiletin and are hypothesized to be demethylated and demethoxylated products of nobiletin. Their MS/MS spectra exhibit fragment ions at *m/z* 329.0674 and 301.0728, corresponding to the consecutive losses of CH_2_O and CO from the protonated metabolites. Metabolite M4 is detected as the protonated molecule [M + H]^+^ at *m/z* 433.1497 (C_22_H_24_O_9_ ), 30 Da heavier than nobiletin. Product ions at *m/z* 403.1018 and *m/z* 373.0808 were observed, consistent with sequential neutral losses of 30 Da (formaldehyde). Consequently, M4 is inferred to be a product of combined oxidation and methylation of nobiletin. M5 (molecular formula C_26_H_28_O_14_ ) generated an [M + H]^+^ ion at *m/z* 565.1558, 162 Da higher than nobiletin, indicating conjugation with glucuronic acid accompanied by demethylation. Its fragment ion at *m/z* 389.1244 resulted from the loss of one molecule of glucose from M5, followed by the loss of one molecule of CH_2_O to yield *m/z* 359.0784. Based on the biosynthetic transformation pattern, accurate mass, characteristic fragment ions, and literature reports, metabolite M5 was tentatively identified as 5′‐methoxynobiletin.

##### 3.1.2.2 Metabolites of Naringenin

The principal metabolic pathways of naringenin include dehydroxylation, hydrogenation, demethylation, ketogenesis, and glucuronidation. Metabolite M6 was detected as the protonated molecule [M + H]^+^ at *m/z* 255.0655, 18 Da lower than naringenin, corresponding to the molecular formula C_15_H_10_O_4_ . This mass shift indicates a single dehydroxylation event. The fragment ions of M6 at *m/z* 227.0714 and *m/z* 199.0771 were generated by the sequential loss of CO from the parent ion, while the fragment ion at *m/z* 237.0569 resulted from the loss of one molecule of H_2_O from the parent ion. A RDA cleavage of M6, following the first ring‐opening pathway, afforded the diagnostic fragment at *m/z* 137.0245. M7 and M8, with the molecular formula C_15_H_16_O_4_ , are 12 Da lower than naringenin and were identified as hydrogenated and dehydroxylated products of naringenin. M9 and M10, with the molecular formula C_15_H_14_O_6_S, are 50 Da higher than naringenin and were inferred to be bis‐demethylated and sulfated products of naringenin. Similarly, M11 and M12, which are 130 Da higher than naringenin, were hypothesized to be demethylated, dehydrogenated, ketone‐formed, and glucuronidated products of naringenin. Their molecular formula is C_20_H_18_O_9_ , and the molecular ion peak [M‐H]^−^ in negative ion mode was observed at *m/z* 401.0885, with sequential losses of CO_2_ yielding fragment ions at *m/z* 357.0638 and *m/z* 313.0709.

##### 3.1.2.3 Metabolites of Hesperetin

Metabolite M13 (C_16_H_12_O_5_ ) exhibited a protonated molecule [M + H]^+^ at *m/z* 285.0762. Its tandem mass spectrum showed a dominant fragment at *m/z* 270.0539, attributable to the loss of a methyl radical (CH_3_ , 15 Da). Subsequent expulsion of CO (28 Da) yielded *m/z* 242.0591, followed by loss of an OH radical (17 Da) to afford *m/z* 225.0562. This metabolite is 18 Da lower than the parent drug hesperetin. Based on the secondary fragment ions, M13 was inferred to be a dehydroxylated product of hesperetin.

#### 3.1.3 Qualitative Method Validation

The validation results for the qualitative analysis of blood components derived from the FAI‐BCT herbal pair are presented as follows: For specificity, as shown in Figure S1, no endogenous interference was observed in the blank matrix within the retention time and accurate mass range of the target compounds. For precision, the results showed that the within‐run and between‐run retention time RSD values of the five reference compounds were ≤ 0.12%, the precision RSD values were ≤ 7.67%, and the accurate mass errors were all within 5 ppm (see Table S1 for details). For matrix effect, the results showed no significant matrix interference, with matrix effect ratios ranging from 91.27% to 108.63% and RSD values ≤ 4.56% (see Table S2 for details). For stability, the results showed that the RSD values of all five reference compounds were ≤ 12.15%, indicating good stability under the tested conditions (storage at room temperature for 4 h, 4°C for 12 h, three freeze–thaw cycles, post‐preparation storage in the autosampler at 4°C for 24 h, and −80°C for 21 days) (see Table S3 for details).

### 3.2 Target Network Pharmacology

#### 3.2.1 Compound Targets of FAI‐BCT

A total of 395 targets were retrieved from the Swiss‐Target‐Prediction database, corresponding to 27 absorbed constituents and their 13 metabolites in FAI‐BCT.

#### 3.2.2 Chronic Gastritis Targets

**FIGURE 6:**
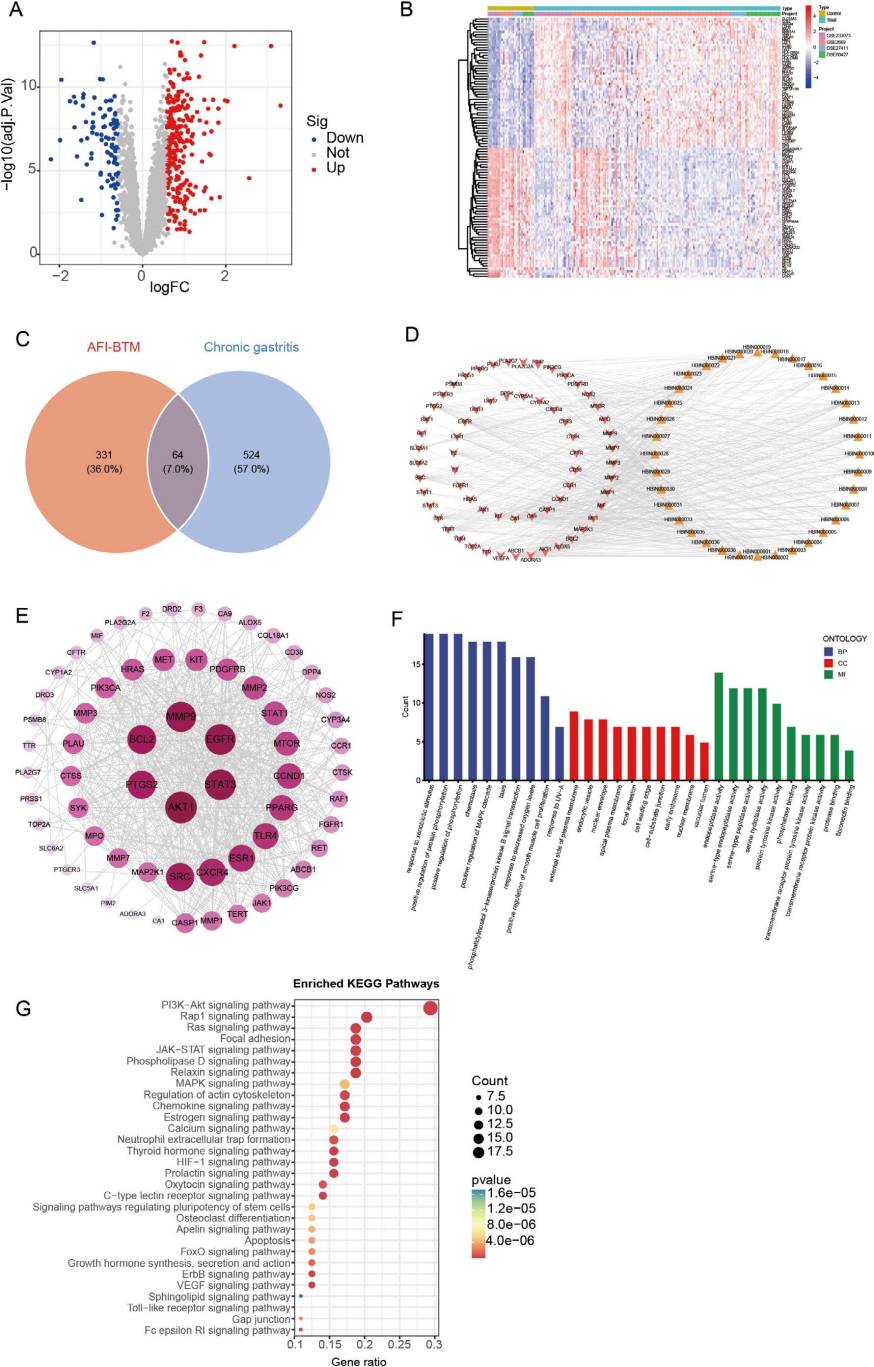
Network pharmacology. (A) Volcano plot; (B) Dendrogram for DEGs; (C) 64 common targets in both chronic gastritis and FAI‐BCT; (D) FAI‐BCT absorbed compounds‐disease targets; (E) PPI network of chronic gastritis and FAI‐BCT targets; (F) GO enrichment analysis; (G) KEGG pathway enrichment analysis of top 30.

Gene expression data for chronic gastritis were obtained from the GEO database (accessions: GSE233973, GSE60427, GSE27411, and GSE2669), comprising 64 normal control samples and 128 gastritis patient samples. PCA was performed to visualize sample distribution patterns post‐correction (Figure S2). A total of 366 differentially expressed genes (DEGs) were identified, with 267 upregulated and 99 downregulated genes (Figure 6A). A heat map of the top 50 differentially expressed genes in 192 samples (sorted by adjusted *p*‐values) is shown in Figure 6B. In parallel, disease‐related targets for chronic gastritis were collected from GeneCards, OMIM, TTD, PharmGKB, and DrugBank. After merging with GEO‐derived targets and removing duplicates, 588 unique disease targets were obtained.

#### 3.2.3 Compound‐Target Interaction Network and Screening of Core Ingredients

Using R software, we identified 64 intersecting targets between FAI‐BCT constituent targets and chronic gastritis‐related targets (Figure 6C). Subsequently, a compound‐target interaction network was constructed with Cytoscape version 3.7.2 (Figure 6D). Compounds with degree centrality ≥ 10 were selected as putative active constituents of FAI‐BCT for chronic gastritis intervention, including: 3′,4′,5,7‐tetramethoxyflavone, 5′‐methoxynobiletin (M5), naringenin, hesperetin, p‐coumaric acid, ferulic acid, and nobiletin.

#### 3.2.4 PPI Network, GO and KEGG Enrichment Analysis

The 64 intersecting targets were imported into the STRING database to construct a protein–protein interaction (PPI) network, followed by visualization using Cytoscape. Node size and color intensity were proportional to degree centrality. The top six targets ranked by degree—namely epidermal growth factor receptor (EGFR), signal transducer and activator of transcription 3 (STAT3), B‐cell lymphoma 2 (BCL2), matrix metalloproteinase‐9 (MMP9), protein kinase B‐α (AKT1), and prostaglandin‐endoperoxide synthase 2 (PTGS2)—were identified as the core targets through which FAI‐BCT exerts its therapeutic effects against chronic gastritis (Figure 6E). GO enrichment analysis was performed on the 64 common targets. The top 10 significantly enriched terms in biological processes (BP), cellular components (CC), and molecular functions (MF) were visualized in a bar chart (Figure 6F). Furthermore, GO analysis indicated that FAI‐BCT is likely to modulate multiple biological processes, including phosphorylation, phosphatidylinositol 3‐kinase/protein kinase B (PI3K/AKT) signaling, protein phosphorylation, responses to hypoxia and xenobiotic stimuli, positive regulation of gene expression, and positive regulation of smooth‐muscle‐cell proliferation. Concurrently, KEGG pathway enrichment analysis of the 64 targets was conducted, with the top 30 significantly enriched pathways visualized in a bubble plot (Figure 6G). Results indicated that FAI‐BCT's anti‐chronic gastritis effects may involve modulation of the PI3K‐Akt signaling pathway, phospholipase D signaling pathway, janus kinase–signal transducer and activator of transcription (JAK–STAT) signaling pathway, and vascular endothelial growth factor (VEGF) signaling pathway.

### 3.3 Molecular Docking and Molecular Dynamics

#### 3.3.1 Molecular Docking

**FIGURE 7:**
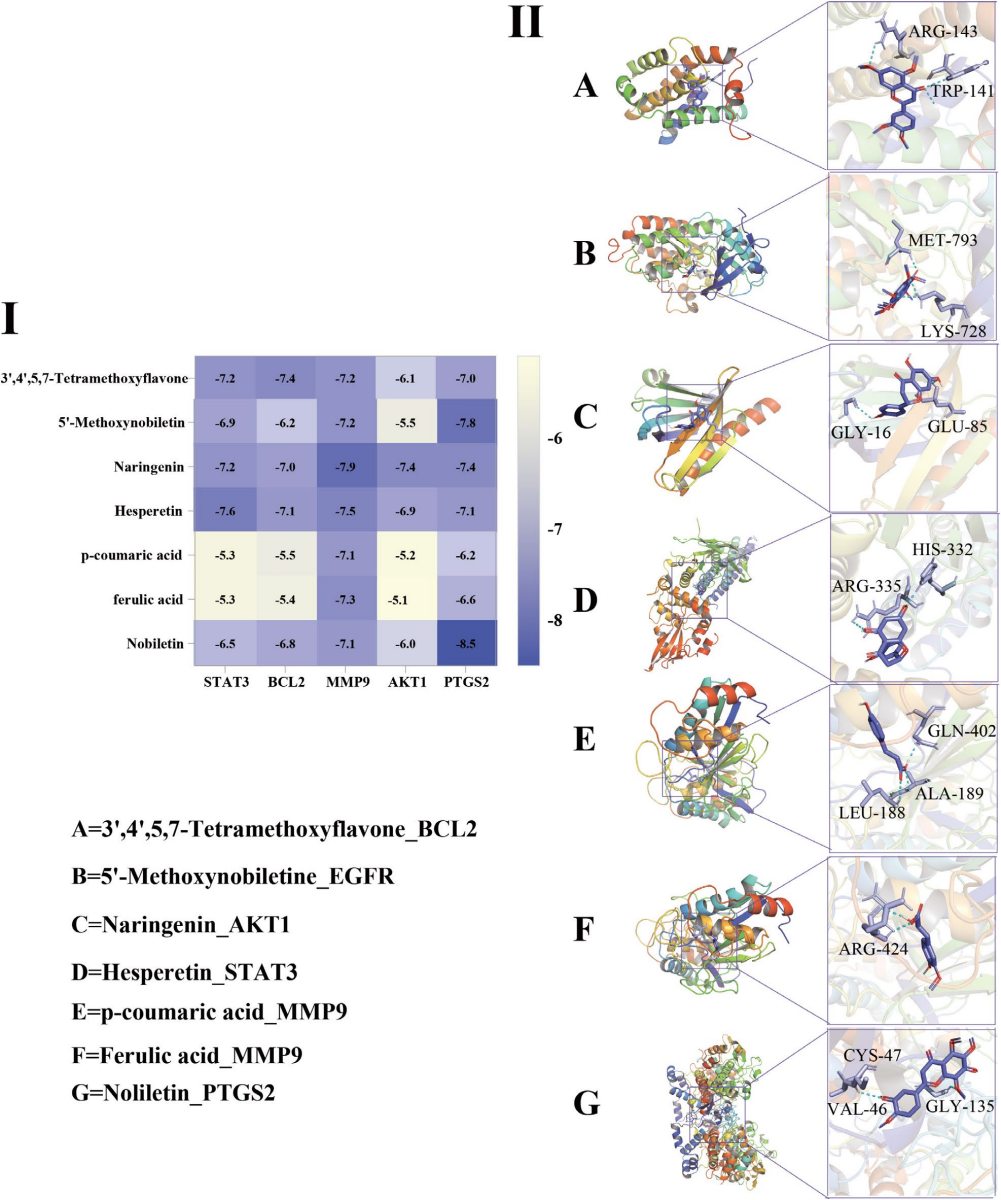
(I) Heat map of molecular docking score. (II) Docking results of binding energy (kJ/mol) of key targets and active compounds of FAI‐BCT herb pair in 3D images: (A) 3′,4′,5,7‐Tetramethoxyflavone_BCL2; (B) 5′‐Methoxynobiletine_EGFR; (C) Naringenin_AKT1; (D) Hesperetin_STAT3; (E) p‐coumaric acid_MMP9; (F) Ferulic acid_MMP9; (G) Noliletin_PTGS2.

Based on the 7 core active constituents and 6 core targets identified through network pharmacology screening, we performed molecular docking and calculated their binding free energy (ΔG). Lower binding free energy values indicate stronger ligand‐target affinity. A lower ΔG value indicates a higher ligand–target affinity; therefore, an effective binding threshold was set at ΔG < −5.0 kJ/mol (Wei et al. 2025). For each ligand‐target pair, the conformation exhibiting the lowest ΔG was selected as the optimal binding mode for subsequent analysis. As shown in Figure 7I (binding energy heatmap), the binding energies of the core components with the key targets were all below −5.1 kJ/mol, with color intensity negatively correlated with binding free energy values. Deep purple regions indicate lower binding free energy (i.e., more stable binding). Figure 7II illustrates the specific molecular docking conformations of complexes formed between each core constituent and its corresponding target with the lowest binding free energy.

#### 3.3.2 Molecular Dynamics Simulations

**FIGURE 8:**
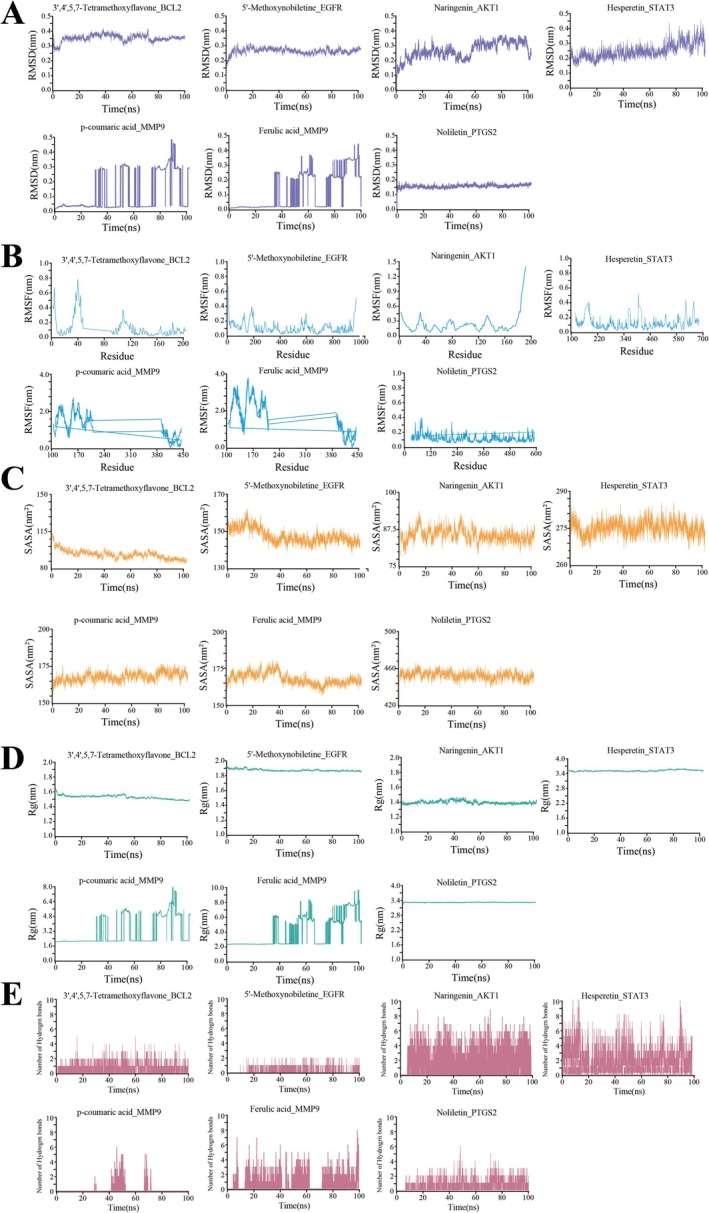
Molecular dynamics simulations. (A) RMSD; (B) RMSF; (C) SASA; (D) Rg; (E) NHB. Figure S1 UPLC‐Q‐TOF‐MS/MS chromatograms of representative reference standards. (A) p‐coumaric acid; (B) ferulic acid; (3) naringenin; (4) hesperetin; (5) nobiletin. Figure S2 PCA Plots of the GEO Dataset Before (A) and After (B) Correction.

To further investigate the stability of protein‐ligand interactions, the top‐ranked complexes from the molecular docking results—specifically 3′,4′,5,7‐Tetramethoxyflavone‐BCL2, 5′‐Methoxynobiletin (M5)‐EGFR, naringenin‐AKT1, hesperetin‐STAT3, p‐coumaric acid‐MMP9, ferulic acid‐MMP9, and nobiletin‐PTGS2—were subjected to 100 ns molecular dynamics simulations. The simulation results were analyzed using RMSD, RMSF, Rg, SASA, and NHB to evaluate the dynamic trajectories of the seven protein‐ligand complexes (details in Figure 8). The RMSD value reflects the conformational fluctuations of a protein and can be used to assess the binding stability between ligands and target proteins. A more stable and balanced curve indicates a more stable binding interaction. Based on the RMSD analysis results in Figure 8A, the simulation trajectories of the p‐coumaric acid‐MMP9 and ferulic acid‐MMP9 complexes exhibited larger fluctuations, suggesting relatively lower structural stability during the simulation process. In contrast, the RMSD curves of the remaining five complexes were converged and stable, indicating that these systems possessed favorable structural stability within the simulation timeframe. The RMSF plot illustrates the flexibility of protein residues throughout the simulation. The abscissa represents the residue number, while the ordinate denotes the fluctuation range of residue positions. Higher values indicate residues with greater flexibility, which may be involved in flexible regions or functional sites of the protein. Lower values denote relatively stable residues. Figure 8B provides insight into the residue fluctuations within the complex, thereby enabling the identification of key fluctuating residues during the molecular dynamics simulation. The SASA describes the interaction between protein molecules and surrounding water molecules, reflecting changes in hydrophilic and hydrophobic properties as well as alterations in surface state. As shown in Figure 8C, the SASA values for all seven protein‐ligand complexes were able to reach an equilibrium state in our simulation results. Rg is commonly used to evaluate the structural compactness of proteins during simulations (Figure 8D). The results demonstrated that the Rg values of the complexes 3′,4′,5,7‐Tetramethoxyflavone‐BCL2, 5′‐Methoxynobiletin (M5)‐EGFR, naringenin‐AKT1, hesperetin‐STAT3, and nobiletin‐PTGS2 remained within a stable fluctuation range throughout the entire simulation period. This indicates that the corresponding proteins maintained favorable overall structural compactness and conformational stability. In contrast, the Rg trajectories of the p‐coumaric acid‐MMP9 and ferulic acid‐MMP9 complexes exhibited pronounced fluctuations, suggesting that their protein structures may have undergone substantial conformational changes or possessed relatively lower stability during the simulation. Hydrogen bonds are among the strongest non‐covalent interactions. The NHB formed during the simulation reflects binding strength, with a higher count denoting greater stability. Monitoring the temporal evolution of hydrogen bond counts within ligand‐protein complexes provides insight into the dynamic characteristics of the binding process. Among the seven ligand‐protein complex systems studied, considerable hydrogen bond interactions were observed in all systems except p‐coumaric acid‐MMP9 (Figure 8E). Based on the comprehensive validation analyses presented above, this study confirms that all five complex systems—3′,4′,5,7‐Tetramethoxyflavone‐BCL2, 5′‐Methoxynobiletin (M5)‐EGFR, naringenin‐AKT1, hesperetin‐STAT3, and nobiletin‐PTGS2—demonstrate significant conformational dynamics stability and potent ligand‐protein interactions.

## 4 Discussion

The therapeutic efficacy of TCM hinges on the in vivo processes of its chemical constituents. Elucidating the material basis of herb pair compatibility, it is essential to systematically characterize the entire process of “absorption into blood→metabolic transformation in vivo→pharmacodynamic effect”. Current research predominantly focuses on “constituent analysis of the raw herbs” and “investigation of the pharmacological mechanisms in vitro”. However, the post‐absorption exposure profile in blood and its temporal changes, as well as the subsequent biotransformation processes such as gut microbial metabolism and hepatic enzyme metabolism, remain insufficiently understood. Therefore, direct analysis of prototype constituents and metabolites present in drug‐containing serum is crucial to identify the genuine “in vivo efficacy material spectrum”, which serves as a key entry point for unveiling the scientific rationale underlying TCM compatibility. In this study, we employed UPLC‐Q‐TOF‐MS/MS technology to investigate the absorbed constituents (prototype compounds and metabolites) of the FAI‐BCT herb pair. A total of 27 prototype components and 13 metabolites absorbed into blood were identified after deducting blank serum. All these compounds were detected in plasma, indicating their absorption into the bloodstream. The prototype compounds primarily included flavonoids, alkaloids, organic acids, and triterpenoids, highlighting that flavonoids are the predominant absorbed constituents and likely the principal bioactive components following oral administration in rats. Previous studies have also demonstrated that flavonoids from Citrus species are major bioactive compounds with antioxidant, anti‐inflammatory, antitumor, and antimicrobial activities (Peluso et al. 2015; Duan et al. 2017; Tong et al. 2018). Metabolic pathways involved predominantly single or combined reactions such as dehydroxylation, demethylation, oxidation, demethoxylation, hydrogenation, methylation, glucuronidation, and sulfation. Based on the identified prototype constituents and their metabolites in serum, a comprehensive analytical database was constructed, encompassing information on molecular formulas, chemical structures, exact masses, mass deviations, characteristic diagnostic fragment ions, and specific metabolic pathways.

Targeted network pharmacology on these absorbed constituents prioritized seven candidate active compounds (3′,4′,5,7‐tetramethoxyflavone, 5′‐methoxynobiletin (M5), naringenin, hesperetin, p‐coumaric acid, ferulic acid, and nobiletin) and six core targets (AKT1, STAT3, EGFR, MMP9, BCL2, and PTGS2) linking the FAI‐BCT herb pair to chronic gastritis pathogenesis. Literature evidence has demonstrated that 5′‐methoxynobiletin (M5) exerts anti‐inflammatory effects by attenuating the expression of phosphorylated p‐p65 NF‐κB and p‐p38 MAPK, thereby disrupting the transcription of inflammation‐related genes (Faqueti et al. 2016, 2020). Naringenin downregulates AKT1, MMP9, and BCL2 to inhibit gastric cell proliferation (Bao et al. 2016; Shin and Shin 2024); Additionally, naringenin exhibits analgesic and anti‐inflammatory effects by suppressing carrageenan‐induced rat paw edema (Chung et al. 2019). Hesperetin blocks pro‐inflammatory signal transduction by inhibiting the phosphorylation of extracellular signal‐regulated kinase 1/2 (ERK1/2) and p38 mitogen‐activated protein kinase (p38 MAPK) and downregulates the Toll‐like receptors 2/4 (TLR2/TLR4)‐myeloid differentiation factor 88 (MyD88) signaling axis, thereby significantly reducing the production of interleukin‐6 (IL‐6), interleukin‐1β (IL‐1β), and tumor necrosis factor‐alpha (TNF‐α) in microglia (Song et al. 2024). Nobiletin exerts gastrointestinal protective effects through a multi‐target mechanism. In colitis models, it downregulates the expression of collagen and pro‐inflammatory cytokines (IL‐6, TNF‐α, CCL2), thereby alleviating inflammation and fibrosis (Hagenlocher et al. 2019). Against gastric injury, nobiletin selectively inhibits arachidonate 5‐lipoxygenase (ALOX5) and PTGS2 to attenuate ethanol‐induced epithelial damage and mitochondrial dysfunction and suppresses *Helicobacter pylori* ‐associated gastric tumorigenesis (Song et al. 2025). Furthermore, nobiletin downregulates ATP‐citrate lyase (ACLY) to induce a lipid‐deprived state and triggers autophagy‐dependent cell death by inhibiting the PI3K/AKT/mTOR pathway, consequently inhibiting gastric cancer cell proliferation (Chen et al. 2024). *p*‐Coumaric acid exerts immunomodulatory and anti‐inflammatory effects by downregulating TNF‐α expression in the synovial tissue of rats with adjuvant‐induced arthritis and reducing circulating immune complex (CIC) levels (Pragasam et al. 2013). Ferulic acid exerts multi‐target anti‐inflammatory effects by inhibiting the expression and activity of key inflammatory mediators such as prostaglandin E2 (PGE2) and TNF‐α and downregulating inducible nitric oxide synthase (iNOS) expression, thereby showing promising potential for the development of anti‐inflammatory drugs (Chaudhary et al. 2019). Collectively, these findings allow us to infer that the FAI‐BCT herb pair exerts therapeutic effects against chronic gastritis through the anti‐inflammatory and anti‐tumor pathways mediated by their core bioactive constituents.

Network pharmacological analysis has identified six core targets that play critical roles in the pathogenesis of chronic gastritis: (1) EGFR, a tyrosine kinase receptor regulating cellular homeostasis, drives malignant transformation of the gastric mucosa through downstream signaling (Silva et al. 2010); (2) STAT3, a central hub of the JAK–STAT signaling pathway, modulates cell growth and inflammation (Zou et al. 2020); (3) BCL2, an anti‐apoptotic oncoprotein, whose activation inhibits apoptosis in gastric cancer cells and thereby promotes gastric tumorigenesis (Ryszczuk et al. 2016); (4) MMP9, which mediates gastric mucosal injury through extracellular matrix degradation (Hsieh et al. 2022); (5) AKT1, a core component of the PI3K/AKT signaling pathway, governs cell survival (Mansouri et al. 2018); (6) PTGS2, an inducible enzyme implicated in both gastritis and gastric carcinogenesis, participates in gastric tumor growth and facilitates malignant transformation, and exhibits distinct methylation profiles in chronic gastritis (Wang et al. 2005). Studies indicate that EGFR and AKT1 serve as shared key biomarkers for distinguishing gastric cancer from chronic gastritis (Mansouri et al. 2018). In children infected with *Helicobacter pylori* , the number of gastric mucosal EGFR and BCL2 protein‐positive cells is significantly upregulated, regardless of the severity of gastritis (mild, moderate, or severe) (Ryszczuk et al. 2016). Published studies have further validated the associations between these components and core targets: naringenin alleviates chronic atrophic gastritis by regulating AKT1 (Lian et al. 2024); hesperetin countered ulcerative colitis through regulation of the JAK2/STAT3/SOCS3 pathway (Elhennawy et al. 2021); and nobiletin reduces gastric epithelial injury by targeting PTGS2 (Song et al. 2025). Collectively, the established associations between active constituents and their specific targets validate the core value of the FAI‐BCT herb pair, warranting further exploration of their mechanisms and application potential.

GO enrichment analysis revealed the multidimensional mechanisms underlying FAI‐BCT intervention in chronic gastritis. GO analysis showed that the core targets were mainly involved in the response to external stimuli, positive regulation of protein phosphorylation, PI3K/AKT signal transduction, and mitogen‐activated protein kinase (MAPK) cascade. These targets were localized to the plasma membrane and endocytic vesicles, while their molecular functions centered on serine‐type endopeptidase and protein tyrosine kinase activity. Similarly, KEGG enrichment analysis indicated that the PI3K‐AKT and Janus kinase/signal transducer and activator of transcription (JAK/STAT) signaling pathways were the major intervention pathways. The PI3K‐AKT pathway mediated by the core targets promotes cell proliferation and inhibits apoptosis, processes that are significantly enhanced during gastric cancer development (Xu et al. 2022; Chen et al. 2020). This is consistent with previous evidence that nobiletin inhibits the proliferation of gastric cancer cells by intervening in the PI3K/AKT/mTOR pathway (Chen et al. 2024). JAK–STAT signaling coordinates inflammatory responses and drives malignant transformation of the gastric mucosa during *Helicobacter pylori* infection (Li et al. 2022; Lin et al. 2025). Thus, FAI‐BCT appears to interrupt the pathological progression from gastritis to gastric cancer through the coordinated modulation of these critical signaling networks.

Molecular docking was performed using the core targets (AKT1, STAT3, EGFR, MMP9, BCL2, and PTGS2) and the core interacting components. The results demonstrated that the core components exhibited favorable binding activity with their corresponding core targets, with binding free energies all below −5.0 kJ/mol. This finding provides a theoretical basis for further experimental validation and supports the potential of these core components as anti‐gastritis agents. Given the inherent limitations of molecular docking in accounting for conformational flexibility and solvent‐induced effects, its predicted optimal binding conformation may not accurately reflect the stable binding state within biological milieu. To rigorously evaluate the dynamic stability of the complexes, we further performed molecular dynamics simulations on the composite systems, integrating nanosecond‐scale trajectory analysis with binding free energy calculations to corroborate the reliability of the predicted interactions (Pang et al. 2024; Yang et al. 2024). All molecular dynamics simulations were conducted using the Gromacs computational platform, we employed molecular dynamics simulations on the optimal binding conformations identified by molecular docking screening for each system. By integrative analysis of multidimensional trajectory parameters—including RMSD, RMSF, Rg, SASA, and NHB—derived from molecular dynamics simulations, we verified that the following complexes exhibit superior conformational dynamical stability and strong intermolecular interactions: 3′,4′,5,7‐tetramethoxyflavone–BCL2, 5′‐methoxynobiletin (M5)–EGFR, naringenin–AKT1, hesperetin–STAT3, and nobiletin–PTGS2. In contrast, the p‐coumaric acid–MMP9 and ferulic acid–MMP9 complexes demonstrated pronounced fluctuations in RMSD and RMSF trajectories, failing to meet the conformational stability criteria and were therefore excluded.

This study has several limitations. Firstly, the findings are based solely on computational simulation predictions and computational validation without experimental verification in vivo. Secondly, the research focused exclusively on serum components, and the bioactivity of metabolites mediated by the gut microbiome warrants further investigation. Finally, the animal model used did not fully replicate the clinical heterogeneity of chronic gastritis, which may limit the relevance of its translational application.

## 5 Conclusions

This study systematically elucidated the multi‐target mechanism of the FAI‐BCT herb pair in preventing and treating chronic gastritis by integrating absorbed bioactive components, targeted network pharmacology, molecular docking, and molecular dynamics simulations. Key findings confirmed that the core pharmacodynamic substances within FAI‐BCT—including 3′,4′,5,7‐tetramethoxyflavone, 5′‐methoxynobiletin (M5), naringenin, hesperetin, and nobiletin—exert therapeutic effects by cooperatively modulating signaling networks associated with chronic gastritis. Molecular dynamics simulations demonstrated the formation of highly stable complexes between these core components and core targets (BCL2, EGFR, AKT1, STAT3, and PTGS2), thus validating the reliability of network pharmacology predictions at the molecular level. Although the present computational models reveal the potential mechanism of action of FAI‐BCT, its clinical translation necessitates further experimental validation through in vitro and in vivo studies to confirm target modulation functionality and evaluate pharmacodynamic efficacy. This study represents the first multi‐dimensional computational simulation system confirming the molecular basis of FAI‐BCT as a therapeutic strategy for chronic gastritis, highlighting its potential as a promising drug candidate with translational medicine value.

## Author Contributions

**Yinghua Ma:** writing – original draft, writing – review and editing, project administration. **Weiwei Xie:** software, data curation. **Jing Song:** formal analysis. **Yabin Qin:** methodology. **Yile Zhao:** investigation, supervision.

## Funding

This work was supported by the Scientific Research Program Project of Hebei Provincial Administration of Traditional Chinese Medicine, 2021209.

## Ethics Statement

The Animal Ethics Committee of Research Ethics Committee of the Second Hospital of Hebei Medical University approved all experiments (No. 2024‐AE065).

## Conflicts of Interest

The authors declare no conflicts of interest.

## Supporting information


**Figure S1:** UPLC‐Q‐TOF‐MS/MS chromatograms of representative reference standards. (A) p‐coumaric acid; (B) ferulic acid; (3) naringenin; (4) hesperetin; (5) nobiletin.

**Figure S2:** PCA Plots of the GEO Dataset Before (A) and After (B) Correction.

**Table S1:** Within‐run and between‐run precision of five reference substance.
**Table S2:** Matrix effects in normal plasma of five reference substance (*n* = 6).
**Table S3:** Stability for five reference substance (*n* = 5).

## Data Availability

The data that support the findings of this study are available from the corresponding author upon reasonable request.
