# Supplementary figures and images for: Unraveling the Bioactive Compounds and Multi‐Target Mechanisms of the Fructus Aurantii Immaturus‐Bambusae Caulis in Taeniam Herb Pair Against Chronic Gastritis: Integrating Identification of Absorbed Constituents, Targeted Network Pharmacology, and Computational Validation
Source: Food Sci Nutr. 2026 Jul 1;14(7):e72016. doi: 10.1002/fsn3.72016 (PMC13320820; doi:10.1002/fsn3.72016)

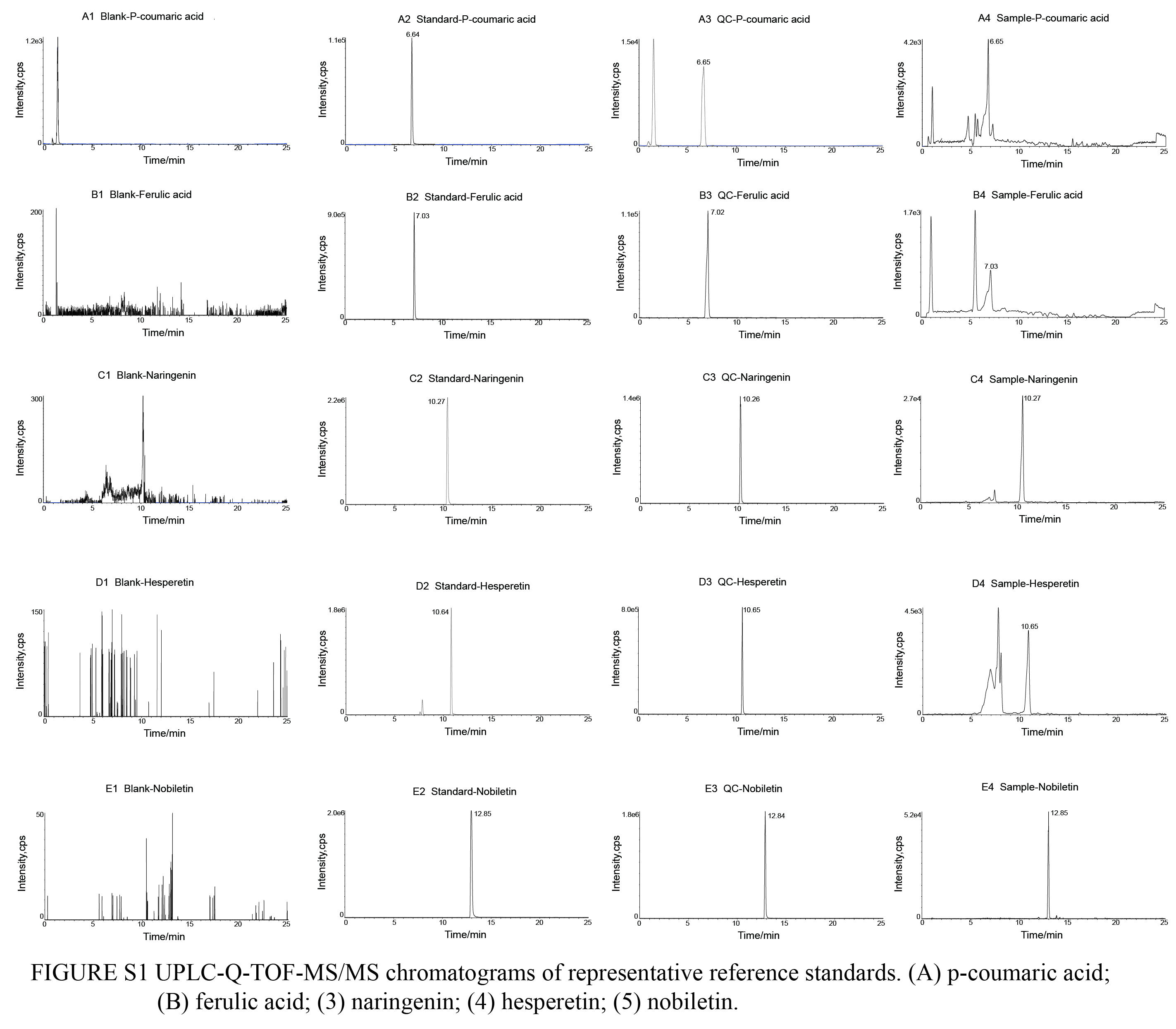

Supplement: Supplementary file 1 — Figure S1: UPLC‐Q‐TOF‐MS/MS chromatograms of representative reference standards. (A) p‐coumaric acid; (B) ferulic acid; (3) naringenin; (4) hesperetin; (5) nobiletin. [file FSN3-14-e72016-s001.tif]

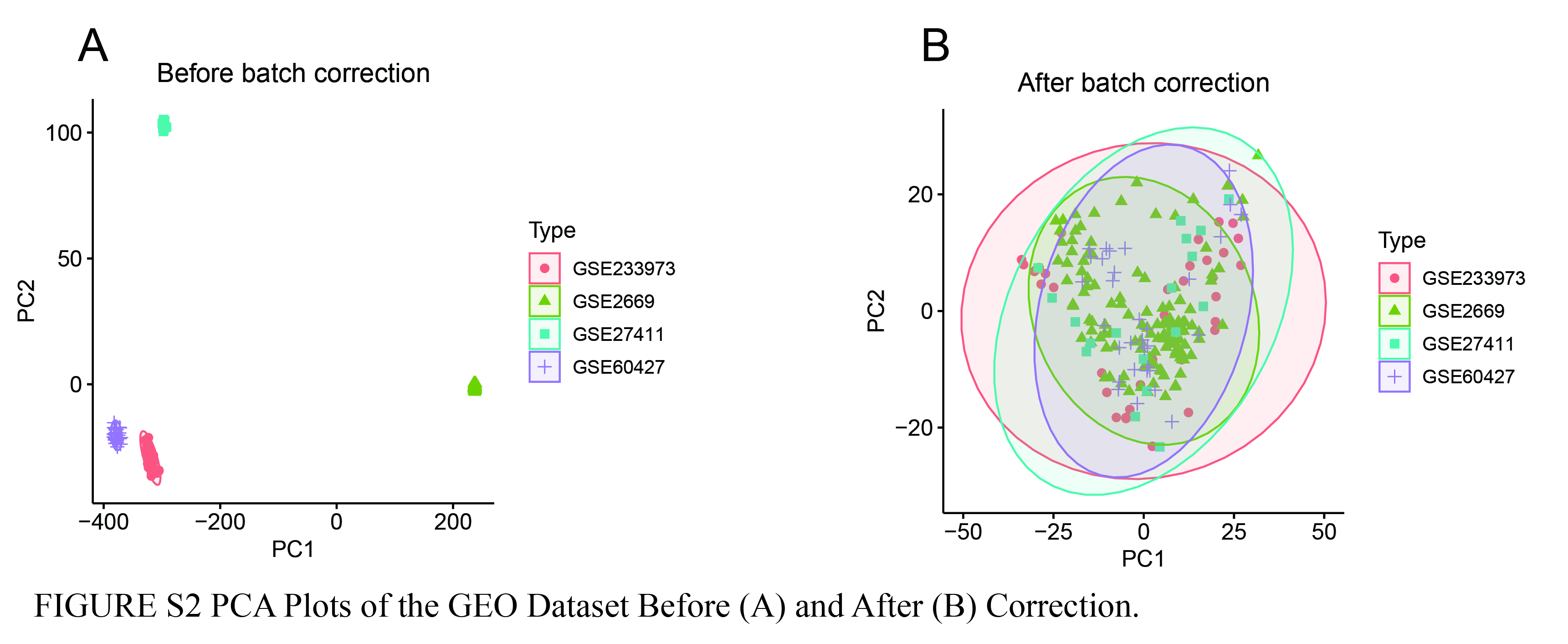

Supplement: Supplementary file 2 — Figure S2: PCA Plots of the GEO Dataset Before (A) and After (B) Correction. [file FSN3-14-e72016-s003.tif]
