# Supplementary material for: Unraveling the Bioactive Compounds and Multi‐Target Mechanisms of the Fructus Aurantii Immaturus‐Bambusae Caulis in Taeniam Herb Pair Against Chronic Gastritis: Integrating Identification of Absorbed Constituents, Targeted Network Pharmacology, and Computational Validation
Source: Food Sci Nutr. 2026 Jul 1;14(7):e72016. doi: 10.1002/fsn3.72016 (PMC13320820; doi:10.1002/fsn3.72016)
Supplement: Supplementary file 3 — Table S1: Within‐run and between‐run precision of five reference substance. Table S2: Matrix effects in normal plasma of five reference substance (n = 6). Table S3: Stability for five reference substance (n = 5). [file FSN3-14-e72016-s002.doc]

**TABLE S1 Within-run and between-run precision of five reference substance.**

| **No.** | **Compound** | **Adduct** | **Theoretical mass concentration/μg·mL^-1^** | **Within-run (*n*=6)** | | | | |  | **Between-run (*n*=18)** | | | | |
| --- | --- | --- | --- | --- | --- | --- | --- | --- | --- | --- | --- | --- | --- | --- |
|  |  |  |  | **Peak area** | **RSD/%** | **Rt/min** | **RSD/%** | **Error/ppm** |  | **Peak area** | **RSD/%** | **Rt/min** | **RSD/%** | **Error/ppm** |
| C2 | p-coumaric acid | [M－H]^－^ | 24.4 | 4.80e6±1.79e5 | 3.73 | 6.65±0.01 | 0.11 | (-0.60)±0.35 |  | 4.78e6±3.23e5 | 6.75 | 6.64±0.01 | 0.11 | (-0.72)±0.28 |
| C4 | Ferulic acid | [M－H]^－^ | 21.4 | 4.75e6±2.24e5 | 4.72 | 7.02±0.00 | 0.07 | (-1.90)±1.12 |  | 4.54e6±2.59e5 | 5.70 | 7.02±0.01 | 0.12 | (-1.59)±0.75 |
| C14 | Naringenin | [M－H]^－^ | 54.0 | 8.06e7±1.77e6 | 2.20 | 10.27±0.01 | 0.10 | (-2.71)±1.28 |  | 8.07e7±1.70e6 | 2.11 | 10.26±0.01 | 0.09 | (-2.17)±1.18 |
| C18 | Hesperetin | [M＋H]^＋^ | 55.9 | 3.00e7±1.22e6 | 4.06 | 10.64±0.00 | 0.04 | (-2.87)±0.60 |  | 2.81e7±2.16e6 | 7.67 | 10.63±0.01 | 0.09 | (-2.92)±0.83 |
| C25 | Nobiletin | [M＋H]^＋^ | 64.0 | 1.02e8±4.23e6 | 4.14 | 12.85±0.01 | 0.06 | (-2.84)±1.17 |  | 1.01e8±5.03e6 | 4.98 | 12.85±0.01 | 0.07 | (-2.74)±0.82 |

**TABLE S2 Matrix effects in normal plasma of five reference substance (*n*=6).**

| **No.** | **Compound** | **Adduct** | **Theoretical mass concentration/μg·mL^-1^** | **Matrix effects/%** | **RSD/%** |
| --- | --- | --- | --- | --- | --- |
| C2 | p-coumaric acid | [M－H]^－^ | 24.4 | 107.53±3.33 | 3.10 |
| C4 | Ferulic acid | [M－H]^－^ | 21.4 | 108.63±3.09 | 2.85 |
| C14 | Naringenin | [M－H]^－^ | 54.0 | 96.71±4.41 | 4.56 |
| C18 | Hesperetin | [M＋H]^＋^ | 55.9 | 91.27±2.34 | 2.56 |
| C25 | Nobiletin | [M＋H]^＋^ | 64.0 | 94.38±2.29 | 2.42 |

**TABLE S3 Stability for five reference substance (*n*=5).**

| **No.** | **Compound** | **Adduct** | **Theoretical mass concentration/μg·mL^-1^** | **Room temperature for 4 h** | **RSD/%** | **4 ◦C for 12 h** | **RSD/%** | **Freeze-thaw**  **Stability** | **RSD/%** | **Autosampler stability** | **RSD/%** | **− 80 ◦C for 3 week** | **RSD/%** |
| --- | --- | --- | --- | --- | --- | --- | --- | --- | --- | --- | --- | --- | --- |
| C2 | p-coumaric acid | [M－H]^－^ | 24.4 | 4.93e6±1.11e5 | 2.26 | 4.72e6±2.68e5 | 5.67 | 4.44e6±2.82e5 | 6.35 | 4.68e6±2.02e5 | 4.32 | 4.29e6±1.56e5 | 4.32 |
| C4 | Ferulic acid | [M－H]^－^ | 21.4 | 4.56e6±2.37e5 | 5.20 | 4.62e6±5.61e5 | 12.15 | 4.34e6±1.70e5 | 3.91 | 4.46e6±1.82e5 | 4.09 | 4.59e6±2.25e5 | 4.09 |
| C14 | Naringenin | [M－H]^－^ | 54.0 | 8.20e7±7.44e5 | 0.91 | 8.370e7±2.37e5 | 2.83 | 7.38e7±1.31e6 | 1.77 | 7.36e7±1.74e6 | 2.37 | 7.39e7±1.40e6 | 2.37 |
| C18 | Hesperetin | [M＋H]^＋^ | 55.9 | 2.67e7±1.40e6 | 5.26 | 2.98e7±1.76e6 | 5.90 | 1.72e7±2.64e5 | 1.54 | 2.23e7±8.52e5 | 3.83 | 1.57e7±7.44e5 | 3.83 |
| C25 | Nobiletin | [M＋H]^＋^ | 64.0 | 9.60e7±5.60e6 | 5.84 | 1.11e8±1.63e6 | 1.47 | 8.63e7±1.89e6 | 2.19 | 9.28e7±5.20e6 | 5.60 | 8.48e7±4.33e6 | 5.60 |
